# Vitamin D Deficiency, Supplementation, and Aging‐Related Morbidity and Mortality Across Two Longitudinal Population Cohorts

**DOI:** 10.1111/acel.70752

**Published:** 2026-10-08

**Authors:** Ariel Israel, Abraham Weizman, Sarah Israel, Shai Ashkenazi, Eytan Ruppin, Eli Magen, Eugene Merzon, Shlomo Vinker

**Affiliations:** ^1^ Department of Epidemiology and Preventive Medicine, School of Public Health, Faculty of Medical & Health Sciences Tel Aviv University Tel‐Aviv Israel; ^2^ Leumit Research Institute Leumit Health Services Tel‐Aviv Israel; ^3^ Research Unit, Faculty of Medical & Health Sciences, Geha Mental Health Center, Felsenstein Medical Research Center and Sagol School of Neurosciences Tel‐Aviv University Tel‐Aviv Israel; ^4^ Department of Clinical Microbiology and Infectious Diseases, Hadassah Medical Center, Faculty of Medicine Hebrew University of Jerusalem Jerusalem Israel; ^5^ Adelson School of Medicine Ariel University Ariel Israel; ^6^ Schneider Children’s Medical Center Petah‐Tikva Israel; ^7^ Translational Research Institute and the Department of Surgery Cedars‐Sinai Medical Center Los Angeles California USA; ^8^ Department of Family Medicine, Faculty of Medical & Health Sciences Tel Aviv University Tel‐Aviv Israel

**Keywords:** aging, causal inference, cohort studies, electronic health records, healthy aging, mortality, vitamin D deficiency

## Abstract

Vitamin D deficiency has been implicated in biological processes linked to aging and organismal resilience, yet its relationship to long‐term systemic aging trajectories remains incompletely characterized. We analyzed longitudinal electronic health record data from two large healthcare systems: Leumit Health Services (LHS), a nationwide Israeli health organization, and TriNetX, a federated US research network. We examined more than 1.67 million serum 25‐hydroxyvitamin D [25(OH)D] measurements from over 468,500 adults in LHS (2009–2020). Longitudinal matched analyses compared severe deficiency (< 10 ng/mL) with sufficiency (> 30 ng/mL) in LHS and were externally validated in 223,175 propensity score‐matched TriNetX pairs; matched cohorts were approximately 54 years old, 65%–67% female, and followed for up to 10 years. In both populations, severe deficiency was associated with increased risks across multiple aging‐related outcomes. All‐cause mortality was higher in LHS (adjusted HR 2.57, 95% CI 2.38–2.79) and TriNetX (HR 1.59, 1.55–1.63). To assess modifiability, we modeled vitamin D supplementation longitudinally using pharmacy‐dispensed purchases calibrated to predict serum 25(OH)D changes over time. Supplementation was independently associated with dose‐dependent reductions across mortality and multiple aging‐related outcomes; for > 10 ng/mL‐equivalent supplementation, mortality HR was 0.72 (0.68–0.76), while showing no corresponding association for skin malignancy. To strengthen causal interpretation, we additionally implemented inverse‐probability‐of‐treatment‐weighted marginal structural Cox models for mortality, which yielded results consistent with conventional adjusted models. Together, these findings support severe vitamin D deficiency as an important and potentially modifiable determinant of systemic aging vulnerability and age‐related multimorbidity in human populations.

## Graphical abstract

Severe vitamin D deficiency was associated with increased mortality and multimorbidity across metabolic, cardiovascular, renal, neurodegenerative, and microvascular domains in two longitudinal population cohorts. Longitudinal supplementation analyses and causal‐framework models supported a potentially modifiable contribution to systemic aging vulnerability.

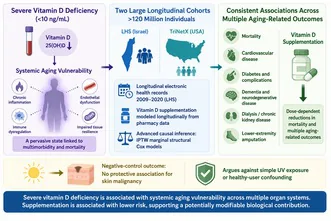

**Abbreviations**
aOR: adjusted odds ratio
BMI: Body Mass Index
BP: blood pressure
CNS: central nervous system
EHR: electronic health records
HR: hazard ratio
ICD‐9: International Classification of Diseases, Ninth Revision
LHS: Leumit Health Services
OR: odds ratio
PO: Per os
PSM: propensity score matching
SES: socioeconomic status
SMD: standardized mean difference

## 1 Introduction

Vitamin D deficiency is widespread worldwide, particularly in populations with limited sun exposure or darker skin pigmentation (Cashman et al. 2016; Hilger et al. 2014; Holick 2007). While its role in bone health is well established, increasing evidence has linked low serum 25‐hydroxyvitamin D [25(OH)D] levels to increased mortality (Gotsman et al. 2012) and a wide range of chronic conditions (Grant et al. 2025), including diabetes (Pittas et al. 2007; Scragg et al. 1995), cardiovascular disease (Acharya et al. 2021; Judd and Tangpricha 2009), retinopathy, chronic kidney disease, foot ulcers (Liu et al. 2024), and neurodegenerative disorders (Afzal et al. 2013; Liu et al. 2011; Usluogullari et al. 2015; Vasdeki et al. 2024). However, despite extensive observational research, the causality of these associations remains disputed. One major challenge is disentangling whether low vitamin D levels are a contributing factor to poor health, or merely a biomarker of existing disease or adverse health behaviors.

Israel, like many countries, has experienced persistently high rates of diabetes‐related complications, including lower‐extremity amputations, with rates particularly elevated compared to other OECD nations (The Commonwealth Fund, n.d.; OECD Reviews of Health Care Quality 2012). At the same time, vitamin D deficiency is common in several demographic subgroups, particularly among individuals with limited sun exposure due to lifestyle, occupational, or cultural practices. Yet, large‐scale longitudinal studies explicitly quantifying the long‐term impact of severe vitamin D deficiency on aging‐related outcomes, and evaluating whether such risks are modifiable, remain scarce.

In this study, we leveraged the electronic health records (EHRs) of Leumit Health Services (LHS) in Israel to assess the long‐term clinical impact of severe vitamin D deficiency (< 10 ng/mL) versus sufficiency (> 30 ng/mL). Using tightly matched cohorts, we examined risks of mortality, diabetes, microvascular and macrovascular complications, and dementia, representing multiple major domains of age‐related morbidity. We then sought to replicate these associations in a large, demographically distinct population through the US‐based TriNetX Research Network. We further evaluated whether pharmacy‐documented vitamin D supplementation was independently and dose‐dependently associated with reduced risk after accounting for baseline 25(OH)D levels. Finally, we applied causal‐inference analyses using inverse‐probability‐of‐treatment‐weighted marginal structural Cox models to evaluate whether the supplementation associations remained robust after accounting for time‐varying treatment selection and measured confounding.

The study comprised four sequential analyses (Figure 1): characterization of the population distribution and seasonality of 25(OH)D in LHS (Analysis A); comparison of severe deficiency and sufficiency in a matched LHS cohort (Analysis B); external validation in the US TriNetX network (Analysis C); and longitudinal assessment of vitamin D supplementation using pharmacy‐dispensing data in LHS (Analysis D).

**FIGURE 1:**
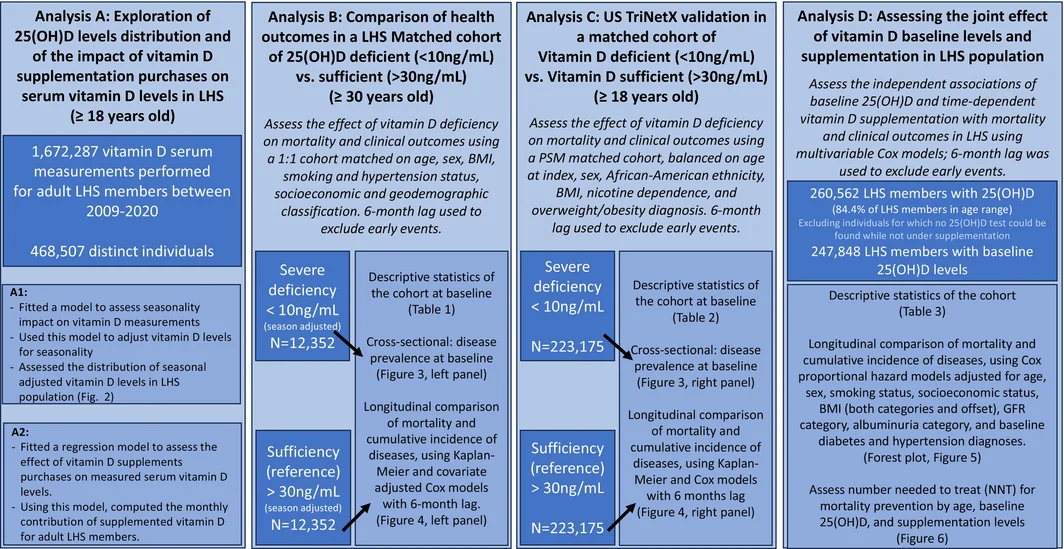
Overview of the analyses performed, showing study populations, age thresholds, exposure definitions, matching, and statistical models used.

## 2 Materials and Methods

### 2.1 Overview

We conducted a retrospective observational study across two healthcare systems (Israel: Leumit Health Services, LHS; United States: TriNetX). Figure 1 summarizes analytic populations, eligibility, inclusion/exclusion criteria, exposure definitions, matching/PSM, and lags.

### 2.2 Participants

- *Analysis A (LHS)*: Adults ≥ 18 years with at least one serum 25(OH)D measurement performed during 2009–2020. These data were used to characterize the population distribution and seasonal variation of 25(OH)D and to model the contribution of pharmacy‐documented vitamin D supplementation to measured serum 25(OH)D levels.
- *Analysis B (LHS)*: Adults ≥ 30 years with a qualifying serum 25(OH)D measurement were eligible for the matched comparison of severe deficiency (< 10 ng/mL) versus sufficiency (> 30 ng/mL). The index date was the first qualifying measurement. The primary longitudinal analyses used a 6‐month lag, with sensitivity analyses using 12‐ and 24‐month landmark lags (Methods S2; Figure S2; Tables S5–S7).
- *Analysis C (TriNetX)*: Adults ≥ 18 years with qualifying serum 25(OH)D measurements were included in the external validation analysis using harmonized outcome definitions and propensity score matching as described below
- *Analysis D (LHS)*: Adults ≥ 30 years with an available baseline 25(OH)D measurement meeting the supplementation‐analysis eligibility criteria were included in the longitudinal analysis of pharmacy‐documented vitamin D supplementation.

### 2.3 Variables

*Covariates (LHS)*: Cox models were fitted after adjustment for age, sex, smoking status, socioeconomic status, BMI (fine categories plus within‐category offset), eGFR category, albuminuria category, baseline diabetes (ICD‐9 diagnosis or HbA1c ≥ 6.5%), baseline chronic kidney disease, hypertension, and systolic blood pressure. A 6‐month lag excluded early events to reduce reverse causation.

*Covariates (TriNetX)*: Propensity score matching was performed using demographic and cardiometabolic covariates available in TriNetX, including age at index, sex, Black or African American demographic category, BMI, nicotine dependence, overweight/obesity, diabetes, hypertension, and HbA1c.

Outcomes included all‐cause mortality and major clinical outcomes, including myocardial infarction, heart failure, dialysis, diabetic retinopathy, foot/toe amputation, dementia, and skin malignancy. Operational definitions and ICD‐9‐CM mappings for the reported LHS outcomes and key diagnosis‐based covariates are provided in Table S4. Skin malignancy was additionally analyzed separately as melanoma and non‐melanoma skin cancer (Table S7).

### 2.4 Data Sources and Measurement

We used de‐identified electronic health records (EHRs) from two distinct cohorts: Leumit Health Services (LHS), a nationwide Israeli health maintenance organization, and the TriNetX US Collaborative Network, which aggregates de‐identified EHRs from over 70 healthcare organizations (HCOs) (Stein et al. 2025). LHS and TriNetX EHR sources included demographics, diagnoses (ICD‐9/ICD‐10 as applicable), laboratory measurements, anthropometric measures, pharmacy dispensing, and mortality data.

Serum 25(OH)D was measured in the LHS central laboratory using standardized commercial assays. Measurements were performed using a DiaSorin platform before 2020 and an Abbott platform from 2020 onward. Both platforms were subject to routine laboratory calibration and quality‐control procedures. Serum 25(OH)D levels were reported in ng/mL units (1 ng/mL = 2.5 nmol/L).

Medical conditions were defined using diagnosis‐code groups and laboratory thresholds, with detailed outcome definitions provided in Table S4.

### 2.5 Bias and Sensitivity Analyses

We mitigated confounding using several complementary approaches: (i) exact matching and multivariable‐adjusted Cox models in LHS analyses, (ii) propensity score matching with balance diagnostics in TriNetX, and (iii) time‐dependent supplementation analyses incorporating longitudinal pharmacy dispensing data. To reduce reverse causation, prevalent events were excluded and the primary longitudinal analyses applied a 6‐month lag. Sensitivity analyses were repeated using 12‐month and 24‐month lags.

To strengthen causal interpretation of the supplementation analysis for all‐cause mortality, we additionally fitted inverse‐probability‐of‐treatment‐weighted (IPTW) marginal structural Cox models using stabilized weights based on prior supplementation and measured covariates. Robust variance estimates were clustered by individual. Full specification of the treatment models, weighting procedure, and landmark sensitivity analyses is provided in Methods S2.

### 2.6 Seasonal Normalization of Serum 25(OH)D Level

25(OH)D levels display marked seasonal variation. To standardize values across measurement dates, we fitted a sinusoidal regression model using log‐transformed 25(OH)D values with first‐ and second‐order sine and cosine terms to model annual periodicity:

$$\log\left(\text{VitaminD}\right)∼\beta_{0}+\beta_{1}\sin t+\beta_{2}\cos t+\beta_{3}\sin2t+\beta_{4}\cos2t$$

where *t* denotes the day of the year in radians. Adjusted values were computed by estimating serum levels at the population trough, using the fitted model to remove seasonal effects.

### 2.7 Data Extraction and Preprocessing

Structured LHS EHR data were extracted using T‐SQL and processed in Python 3.11 using anonymized study identifiers. Baseline covariates were defined at the index date.

### 2.8 Matched Cohort Construction (LHS)

To evaluate the long‐term impact of severe deficiency, we constructed a 1:1 matched cohort comparing individuals with seasonally normalized, nadir‐equivalent 25(OH)D levels < 10 ng/mL with those with levels > 30 ng/mL (see Seasonal Normalization). Matching was exact on age category, sex, smoking status, BMI category, blood‐pressure category, ethnic sector (general, Arab, ultra‐Orthodox), socioeconomic status, and year of LHS enrollment. The sufficiency group (> 30 ng/mL) served as the reference category in subsequent analyses.

### 2.9 Outcome Discovery in LHS

Approximately 300 common medical conditions, defined using validated ICD‐9 code groups curated by the Leumit Research Institute, were screened in the matched cohort. Cox proportional hazards models were used to compare cumulative incidence between groups, adjusting for covariates. False discovery rate (FDR) control was applied to identify conditions with significant differences.

### 2.10 TriNetX Validation Cohort

For external validation, we compared TriNetX patients with 25(OH)D < 10 ng/mL to those with > 30 ng/mL. Propensity score matching was performed using demographic and cardiometabolic covariates available in TriNetX, including age at index, sex, Black or African American demographic category, BMI, nicotine dependence, overweight/obesity, diabetes, hypertension, and HbA1c. Post‐matching balance was assessed using standardized mean differences. Conditions identified as FDR‐significant in LHS were validated using harmonized definitions in TriNetX with a 6‐month lag to exclude early events.

### 2.11 Vitamin D Supplementation Estimation

All vitamin D‐containing products dispensed through LHS pharmacies were assigned a total vitamin D content according to labeled concentration and package size. We estimated the serum contribution of supplementation using a two‐stage model. First, log‐transformed serum 25(OH)D was modeled from demographic, anthropometric, socioeconomic, temporal, and seasonal variables. Second, residual 25(OH)D was regressed on lagged vitamin D purchases aggregated over monthly, quarterly, and yearly intervals. The estimated coefficients were subsequently used to derive a longitudinal serum‐equivalent supplementation exposure. Full details of exposure construction and calibration are provided in Methods S1, with fitted coefficients reported in Tables S2 and S3.

Although clinical factors such as impaired renal function may influence vitamin D metabolism, no clinical or demographic variables were included in the supplementation model itself to minimize potential reverse causation.

### 2.12 Survival Analysis Using Time‐Dependent Supplementation

We used time‐dependent Cox proportional hazards models to evaluate supplementation effects on mortality and clinical outcomes. Person‐time was split into monthly intervals. Models jointly included baseline 25(OH)D categories and time‐dependent supplementation categories and were adjusted for demographic, metabolic, renal, and cardiovascular covariates. Detailed construction of the longitudinal exposure and model specification is provided in Methods S1.

In MSM/IPTW sensitivity analyses, weighted Cox proportional hazards models were fitted using cumulative stabilized inverse‐probability weights to estimate associations under a pseudo‐population in which measured confounding of supplementation assignment was reduced. Robust sandwich variance estimation clustered by individual was used in all weighted analyses.

### 2.13 Absolute Risk Reduction and Number Needed to Treat (NNT)

Ten‐year absolute risk reductions and corresponding numbers needed to treat (NNTs) were derived from adjusted survival projections under each supplementation category using a standardized reference covariate profile. Detailed calculations are provided in Methods S2.

### 2.14 Statistical Analyses

Categorical variables were compared using Fisher's exact test. Continuous variables were compared using two‐sample *t*‐tests. Logistic regression was used to estimate odds ratios (ORs) with 95% confidence intervals (CIs). Longitudinal outcomes were analyzed using Cox proportional hazards models. Kaplan–Meier curves were used to visualize survival. Death (for nonfatal outcomes), and disenrollment were treated as censoring events in all models. Given the strong association between vitamin D deficiency and mortality itself, competing‐risk models would likely underestimate rather than inflate relative hazards for nonfatal outcomes.

All analyses were performed using R 4.4 and Python 3.11.

### 2.15 Generative AI Statement

ChatGPT was used to assist with language refinement for improved clarity and flow, and to provide support with coding tasks related to data analysis scripts. All content was reviewed and verified by the authors to ensure accuracy and integrity.

### 2.16 Ethics

All analyses of Leumit Health Services (LHS) data were performed retrospectively on fully anonymized electronic health records under institutional review board approval (Leumit Health Services IRB approval no. LEU‐0020‐24), with a waiver of informed consent granted because the study posed minimal risk and used de‐identified data.

Analyses using the US TriNetX Research Network were conducted on de‐identified aggregate data provided under data‐use agreements with participating healthcare organizations and were therefore not considered human‐subjects research; these analyses were compliant with the US Health Insurance Portability and Accountability Act (HIPAA) and local institutional guidelines.

### 2.17 Data Availability

Individual‐level patient data from LHS are not publicly available due to privacy and regulatory restrictions. Aggregated data supporting the findings of this study may be provided to qualified researchers upon reasonable request, subject to approval by the LHS Institutional Review Board.

Data from the TriNetX network are available to institutions that are authorized by the TriNetX collaboration network, through direct request to TriNetX (https://www.trinetx.com).

## 3 Results

### 3.1 Analysis A: Exploration of Vitamin D Distribution and of the Impact of Vitamin D Supplementation Purchases on Serum 25(OH)D Levels in LHS

Serum 25(OH)D showed pronounced seasonal variation, peaking in late summer and reaching a nadir in mid‐February (Figure 2A; Table S1). Applying the seasonal‐normalization model yielded nadir‐equivalent measurements (Figure 2B), from which the population distribution was derived (Figure 2C). Severe deficiency (< 10 ng/mL) was substantially more common in females than males.

**FIGURE 2:**
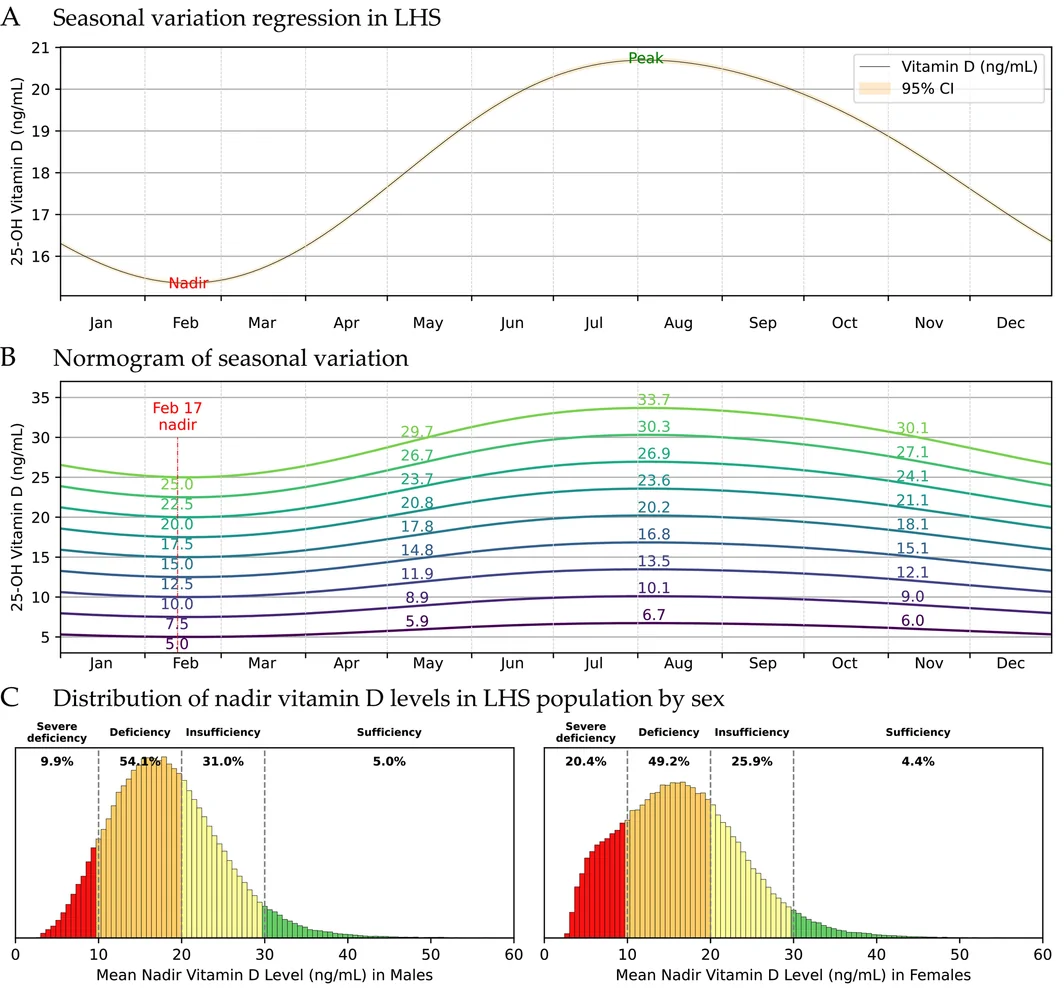
Seasonal variation and population distribution of serum 25(OH)D in LHS (Analysis A). (A) Modeled seasonal fluctuation in serum 25(OH)D levels across the calendar year in the Leumit Health Services (LHS) population, showing a sinusoidal pattern with a nadir in mid‐February and peak in late August. The 95% confidence interval is shown but appears nearly indistinguishable due to the large population size. (B) Normogram translating observed 25(OH)D levels to their expected nadir values based on date of measurement, enabling standardization of vitamin D status across seasons. (C) Sex‐stratified distribution of mean nadir‐adjusted vitamin D levels in the LHS population. Severe deficiency (< 10 ng/mL) is substantially more common in females (20.4%) than in males (9.9%), whereas vitamin D sufficiency (> 30 ng/mL) is uncommon in both sexes.

### 3.2 Analysis B: LHS Matched Cohort of 25(OH)D Deficient (< 10 ng/mL) Versus Sufficient Individuals—Baseline

To evaluate the clinical consequences of severe vitamin D deficiency, we constructed a matched cohort comparing individuals with severe deficiency to those with sufficiency (flowchart in Figure S1). The index date was defined as the first recorded serum 25(OH)D measurement for each individual from 2009 onward. Using the previously fitted seasonal‐correction model, we calculated a day‐of‐year–adjusted, nadir‐equivalent 25(OH)D level for each individual based on the index measurement and classified participants into two exposure groups: severely deficient (< 10 ng/mL) and sufficient (> 30 ng/mL).

Table 1 presents the 12,352 severely deficient participants and 12,352 matched sufficient controls. As expected from the matching design, the two groups were well balanced across all matching variables, with negligible standardized mean differences (SMD < 0.01 for age, BMI, weight and blood pressure measures). The matched groups (mean age 54.4 years; 67% female) showed no meaningful differences in the matched variables.

**TABLE 1:** Demographic and clinical characteristics of the LHS matched cohort at baseline (Analysis B).

	Deficiency 25(OH)D below 10 ng/mL	Sufficiency 25(OH)D above 30 ng/mL (reference)	*p*	Odds ratio	SMD
*N*	12,352	12,352			
Age (years)	54.4 ± 14.8	54.5 ± 14.6	0.625		−0.006
Sex					
Female	8278 (67.0%)	8278 (67.0%)	1.000	1.00 [0.95 to 1.05]	
Male	4074 (33.0%)	4074 (33.0%)	1.000	1.00 [0.95 to 1.05]	
Weight (kg)	75.8 ± 16.8	75.7 ± 16.6	0.833		0.003
Height (cm)	165 ± 9	165 ± 10	0.001		−0.044
Body Mass Index (BMI) (kg/m^2^)	28.0 ± 5.7	28.0 ± 5.7	0.508		0.009
< 18.5 Underweight	182 (1.68%)	191 (1.80%)	0.497	0.93 [0.75 to 1.15]	
18.5–24.9 Normal	3280 (30.22%)	3248 (30.64%)	0.514	0.98 [0.92 to 1.04]	
25–29.9 Overweight	3866 (35.62%)	3734 (35.23%)	0.549	1.02 [0.96 to 1.08]	
≥ 30 Obese	3524 (32.47%)	3427 (32.33%)	0.827	1.01 [0.95 to 1.07]	
~missing~	1500 (12.14%)	1752 (14.18%)			
Smoking status					
Non‐smoker	9347 (79.22%)	9657 (79.84%)	0.235	0.96 [0.90 to 1.03]	
Past smoker	213 (1.81%)	254 (2.10%)	0.102	0.86 [0.71 to 1.03]	
Smoker	2239 (18.98%)	2184 (18.06%)	0.069	1.06 [1.00 to 1.14]	
~missing~	553 (4.48%)	257 (2.08%)			
Blood pressure (mmHg)					
Systolic	126 ± 19	125 ± 18	0.001		0.004
Diastolic	76.1 ± 10.0	76.0 ± 9.6	0.515		0.008
~missing~	7 (0.057%)	5 (0.040%)			
Hypertension status					
Stage 1	2419 (19.6%)	2402 (19.5%)	0.785	1.01 [0.95 to 1.08]	
Stage 2	2074 (16.8%)	2043 (16.5%)	0.597	1.02 [0.95 to 1.09]	
Ethnic group					
Arab	2215 (17.9%)	2215 (17.9%)	1.000	1.00 [0.94 to 1.07]	
General	7976 (64.6%)	7976 (64.6%)	1.000	1.00 [0.95 to 1.05]	
Jewish ultra‐orthodox	2161 (17.5%)	2161 (17.5%)	1.000	1.00 [0.94 to 1.07]	
Socio‐economic status (1–20)	8.39 ± 3.68	8.57 ± 3.53	< 0.001		−0.051
~missing~	417 (3.38%)	417 (3.38%)			

*Note:* This descriptive table summarizes baseline characteristics of the 1:1 matched LHS cohort comparing individuals with severe vitamin D deficiency (< 10 ng/mL) and sufficiency (> 30 ng/mL), with calendar adjustment applied to 25(OH)D (see Methods). Matching was performed on age category, sex, BMI category, smoking status, hypertension stage, socioeconomic status, ethnic sector, and year of LHS enrollment; variables used for exact matching have similar counts across groups by design. The sufficiency group (> 30 ng/mL) serves as the reference category for comparisons; “reference” denotes the a priori comparison category and does not imply majority prevalence. Cohort characteristics are reported at the index date (the date of the first qualifying 25(OH)D measurement during the study period). Baseline covariates were taken as of the index date (using the closest pre‐index measurement when available, and the first post‐index measurement otherwise). Continuous variables are presented as mean ± SD; categorical variables as *n* (%). “missing” indicates no available baseline value for that variable. Percentages for categories are calculated out of the non‐missing values. *p* values are descriptive only and not used for inference or balance decisions in this matched design. For continuous variables, two‐sample *t*‐tests were used; for categorical variables, Fisher's exact test. Odds ratios (ORs) are unadjusted and compare the odds of being in the Deficiency group versus the Sufficiency (reference) group for the listed level (95% CI in brackets). For multi‐level variables, the OR shown is for each level versus all other levels of that variable. SMD, standardized mean difference (|SMD| < 0.10 generally indicates negligible imbalance).

We examined baseline clinical associations with severe vitamin D deficiency in the LHS matched cohort (Figure 3A). Despite rigorous matching on demographic and metabolic factors, individuals with severe deficiency showed markedly higher baseline prevalence of chronic conditions. Elevated odds were observed for diabetes‐related and cardiovascular outcomes, including foot/toe amputation (OR 4.01, 95% CI [2.04 to 8.62], *p* < 10^−5^), diabetes mellitus (OR 1.23, 95% CI [1.15 to 1.31], *p* < 10^−9^), diabetic retinopathy (OR 1.69, 95% CI [1.45 to 1.99], *p* < 10^−10^), dementia (OR 1.89, 95% CI [1.53 to 2.33], *p* < 10^−9^), cerebrovascular accident (CVA) (OR 1.79, 95% CI [1.50 to 2.14], *p* < 10^−10^), and myocardial infarction (OR 1.40, 95% CI [1.25 to 1.57], *p* < 10^−8^). In contrast, the odds of skin malignancy were markedly reduced (OR 0.44, 95% CI [0.31 to 0.61], *p* < 10^−6^), suggesting lower cumulative skin ultraviolet (UV) exposure in the group with low 25(OH)D serum levels.

**FIGURE 3:**
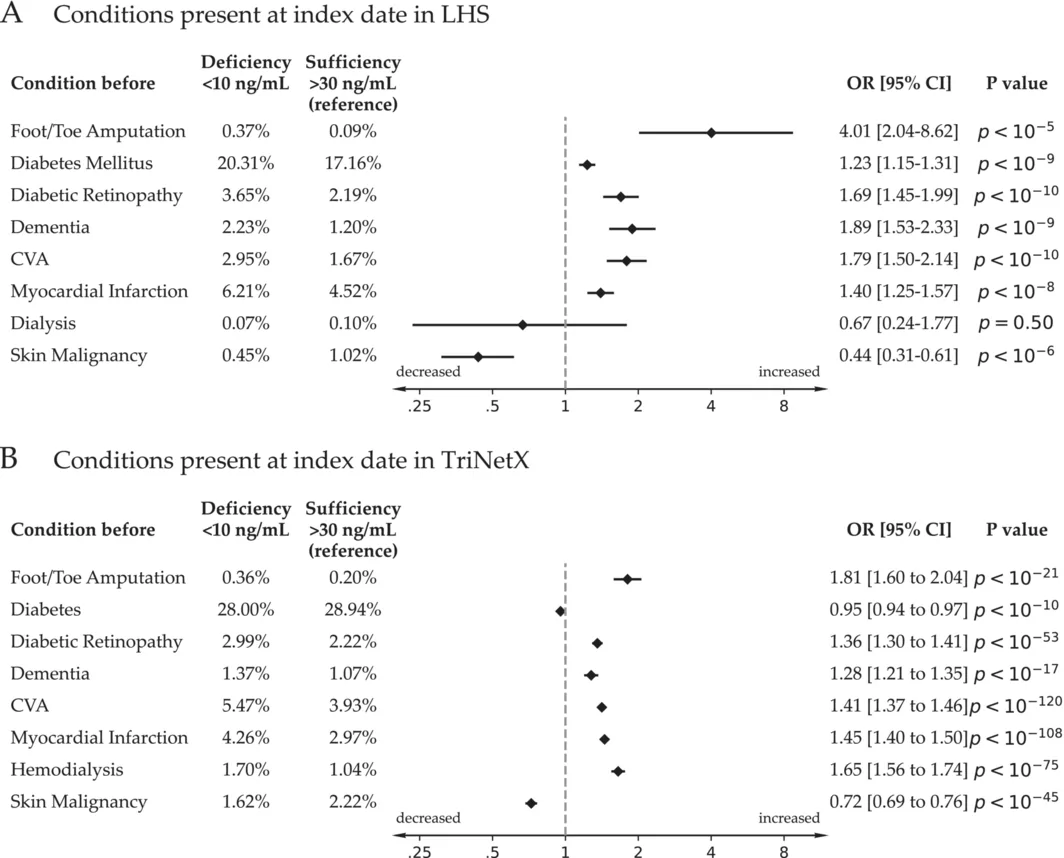
Cross‐sectional baseline prevalence of chronic conditions by vitamin D status in LHS (Analysis B) and TriNetX (Analysis C). (A) Odds ratios (OR) and 95% confidence intervals for preexisting conditions at cohort entry in the Leumit Health Services (LHS) matched cohort, comparing individuals with severe vitamin D deficiency (< 10 ng/mL) to those with sufficient levels (> 30 ng/mL). (B) Corresponding analysis from the TriNetX propensity‐matched cohort. Foot/toe amputation, diabetic retinopathy, dementia, cerebrovascular accident, and myocardial infarction were consistently more prevalent in the vitamin D‐deficient groups across both datasets. Diabetes mellitus was more prevalent in the deficient LHS group but was slightly less prevalent in the propensity‐score‐matched TriNetX cohort, where diabetes was included among the matching covariates. In contrast, skin malignancy was significantly less common among individuals with low vitamin D, likely reflecting reduced cumulative UV exposure. These robust and replicated imbalances underscore the biological relevance of vitamin D status beyond demographic and behavioral confounding. CVA, cerebrovascular accident.

### 3.3 Analysis C: US TriNetX Matched Cohort of 25(OH)D Deficient (< 10 ng/mL) Versus Sufficient Individuals—Baseline

To validate these findings, we performed a similar analysis in an independent population using TriNetX, a global federated health research network that aggregates electronic health records from more than 70 healthcare organizations covering over 120 million patients. Eligible members of the US network had a documented serum 25(OH)D measurement obtained within 1 year of a recorded BMI value. Table 2 presents the clinical characteristics of the cohort at baseline, showing well balanced propensity‐matched TriNetX groups and near‐identical distributions of age (mean ~ 54 years), sex (≈65% female), cardiometabolic risk factors, and laboratory measures.

**TABLE 2:** Demographic and clinical characteristics of the US‐TriNetX matched cohort at baseline (Analysis C).

	Severe deficiency 25(OH)D < 10 ng/mL	Sufficiency 25(OH)D > 30 ng/mL (reference)
*N* before matching	224,419	2,117,525
*N* after matching	223,175	223,175
Age at index (years)	54.4 ± 14.8	54.5 ± 14.6
Age current (years)	60.6 ± 14.1	59.6 ± 13.9
Sex		
Female	65.2%	65.5%
Male	34.8%	34.5%
Smoking status (nicotine dependence)	19.7%	19.8%
Black or African American	52.0%	53.9%
Essential hypertension	32.4%	32.6%
Diabetes Mellitus	28.2%	29.4%
Hemoglobin A1c (%)	6.4 ± 1.9	6.3 ± 1.5
0–5.7	30.1%	30.1%
5.7–6.5	28.5%	29.8%
6.5–7.5	15.2%	15.8%
≥ 7.5	16.0%	16.5%
Fasting serum glucose (mg/dL)	113.4 ± 53.4	109.5 ± 45.3
≥ 100	72.0%	68.3%

*Note:* This table summarizes baseline characteristics of the 1:1 propensity‐score‐matched US‐TriNetX cohort, comparing individuals with severe vitamin D deficiency (< 10 ng/mL) and sufficiency (> 30 ng/mL) defined by their baseline serum 25(OH)D measurement, which served as the index date. All included participants had at least one BMI measurement recorded within 1 year prior to the index date. Propensity score matching was performed using demographic and cardiometabolic covariates available in TriNetX, including age at index, sex, Black or African American demographic category, BMI, nicotine dependence, overweight/obesity, diabetes, hypertension, and HbA1c. The table additionally presents selected clinical and laboratory characteristics to demonstrate post‐matching comparability. The matched cohorts were well balanced across these covariates, demonstrating very similar distributions of age (mean ~ 54.4 years), sex (≈ 65% female), cardiometabolic risk factors, and laboratory measures at baseline. More than half of the cohort participants self‐identified as Black or African American, reflecting the demographic distribution of severe vitamin D deficiency in the TriNetX network and its matched comparison group.

Baseline clinical associations in TriNetX were directionally consistent with those observed in LHS (Figure 3B).

Nevertheless, the TriNetX cohort showed the same pattern of higher odds of chronic conditions at baseline. Notably, the deficient group had significantly higher rates of diabetes‐related and cardiovascular complications, including foot/toe amputation (OR 1.81, 95% CI [1.60–2.04], *p* < 10^−21^), diabetic retinopathy (OR 1.36 [1.30–1.41], *p* < 10^−53^), dementia (OR 1.28 [1.21–1.35], *p* < 10^−17^), CVA (OR 1.41 [1.37–1.46], *p* < 10^−120^), myocardial infarction (OR 1.45 [1.40–1.50], *p* < 10^−108^), and hemodialysis (OR 1.65 [1.56–1.74], *p* < 10^−75^). In contrast, the odds of skin malignancy were significantly lower in the vitamin D‐deficient group (OR 0.72, 95% CI [0.69–0.76], *p* < 10^−45^), again suggesting lower cumulative ultraviolet skin exposure among individuals with low 25(OH)D levels.

### 3.4 Longitudinal Impact of 25(OH)D Deficiency in the LHS and TriNetX Cohorts

To evaluate the longitudinal impact of vitamin D deficiency, we conducted survival analyses excluding individuals who had the outcome of interest at baseline or who developed it within a predefined lag period of 6 months of the baseline measurement. Both cohorts were then followed longitudinally for up to 10 years from the index date, applying a six‐month safety lag. Adjusted Cox models were fitted as described in Methods; Figure 4 shows the corresponding survival curves and effect estimates for LHS and TriNetX.

**FIGURE 4:**
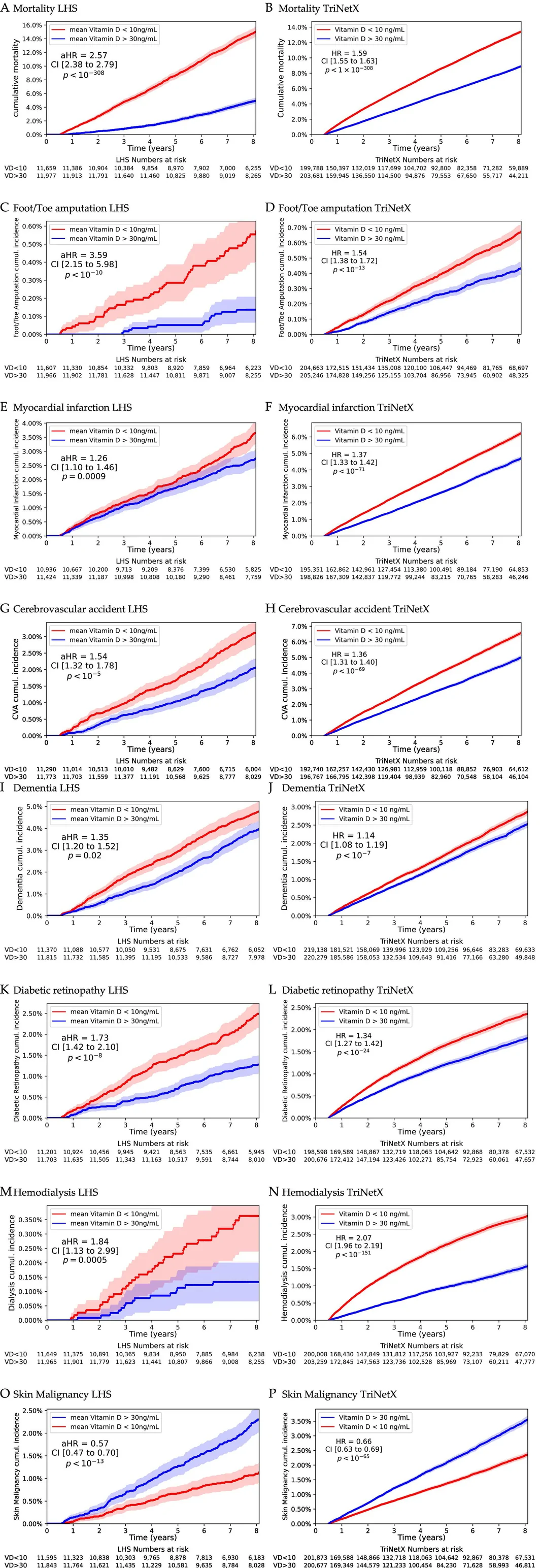
Longitudinal risk of mortality and incident disease by vitamin D status in LHS (left‐panel) and TriNetX (right‐panel). Kaplan–Meier survival and cumulative incidence curves for matched cohorts comparing individuals with baseline serum 25(OH)D < 10 ng/mL versus > 30 ng/mL across two independent cohorts: Leumit Health Services (LHS, left panels) and TriNetX (right panels). Time is years since the index (first qualifying 25(OH)D test). Numbers at risk are shown below each plot. Only incident events occurring ≥ 6 months after the index date were included; individuals with the outcome before or during the initial 6‐month period were excluded from the corresponding analysis. Panels: (A, B) All‐cause mortality; (C, D) Foot/toe amputation; (E, F) Myocardial infarction; (G, H) Cerebrovascular accident; (I, J) Dementia; (K, L) Diabetic Retinopathy; (M, N) Hemodialysis initiation; (O, P) Skin malignancy. LHS (left): Adjusted hazard ratios (aHRs), 95% CI, and *p* values are from Cox models fit in the 1:1 exact‐matched cohort (Table 1) and further adjusted for age (continuous), sex, socioeconomic status (fine categories), baseline diabetes (ICD‐9 diagnosis or HbA1c > 6.5), smoking status (never, past, current, or missing), BMI (fine categories plus an offset from the category lower boundary), kidney function (GFR categories), and albuminuria (urinary albumin/creatinine categories), hypertension, and systolic BP. The reference category is vitamin D sufficiency (> 30 ng/mL). TriNetX (right): Hazard ratios (HRs), 95% CIs, and *p* values are from Cox models in 1:1 propensity‐score‐matched cohorts (Table 2). Propensity‐score matching used demographic and cardiometabolic covariates available in TriNetX, as described in Methods. A 6‐month lag was applied as above. The reference category is vitamin D sufficiency (> 30 ng/mL).

#### 3.4.1 LHS Cohort

- *All‐cause mortality*: cumulative 8‐year incidence ≈ 14% in the deficient group versus ≈ 8% in controls; aHR = 2.57 (95% CI [2.38–2.79], *p* < 10^−308^)
- *Foot/Toe amputation*: aHR = 3.59 ([2.15–5.98], *p* < 10^−10^)
- *Myocardial infarction*: aHR = 1.26 ([1.10–1.46], *p* = 0.0009)
- *Cerebrovascular accident*: aHR = 1.54 ([1.32–1.78], *p* < 10^−5^)
- *Dementia*: aHR = 1.35 ([1.20–1.52], *p* = 0.02)
- *Diabetic retinopathy*: aHR = 1.73 ([1.42–2.10], *p* < 10^−8^)
- *Dialysis*: aHR = 1.84 ([1.13–2.99], *p* = 0.0005)
- *Skin malignancy*: consistently lower in the deficient group (aHR = 0.57 [0.47–0.70], *p* < 10^−13^)

#### 3.4.2 TriNetX Cohort

- *All‐cause mortality*: HR = 1.59 (95% CI [1.55–1.63], *p* < 10^−308^)
- *Foot/toe amputation*: HR = 1.54 ([1.38–1.72], *p* < 10^−13^)
- *Myocardial infarction*: HR = 1.37 ([1.33–1.42], *p* < 10^−71^)
- *Cerebrovascular accident*: HR = 1.36 ([1.31–1.40], *p* < 10^−69^)
- *Dementia*: HR = 1.14 ([1.08–1.19], *p* < 10^−7^)
- *Diabetic retinopathy*: HR = 1.34 ([1.27–1.42], *p* < 10^−24^)
- *Hemodialysis*: HR = 2.07 ([1.96–2.19], *p* < 10^−151^)
- *Skin malignancy*: significantly lower in the deficient group, HR = 0.66 ([0.63–0.69], *p* < 10^−65^)

In both cohorts, the divergence in risk between the deficient and sufficient groups increased over time, even after excluding events that occurred prior to the index date or during the initial six‐month lag period. The longitudinal associations remained materially unchanged when the post‐index lag was extended from 6 months to 12 months (Figure S2) and 24 months (Table S5), with preservation of the direction and magnitude of the principal associations in both LHS and TriNetX.

### 3.5 Modeling Vitamin D Supplementation Impact on 25(OH)D Levels

The baseline model explained 25.4% of the variance in serum 25(OH)D (Table S2). Lagged pharmacy‐documented supplementation explained an additional 9.3% of residual variance, with the largest contributions from purchases closest to the measurement date (Table S3). Together, supplementation variables explained an additional 9.3% of the variance in 25(OH)D levels not captured by baseline characteristics, demonstrating a quantifiable effect of supplement use on serum 25(OH)D levels.

### 3.6 Analysis D: Assessing the Independent Effect of Vitamin D Supplementation

Of 260,562 LHS members aged ≥ 30 years with a 25(OH)D measurement, 247,848 met the baseline exposure requirements for the supplementation analysis (Table 3). Joint time‐dependent models of baseline 25(OH)D and supplementation were fitted as described in Methods (Figure 5).

**TABLE 3:** Demographic and clinical characteristics of the Leumit Health Services (LHS) longitudinal cohort used for analysis of vitamin D supplementation (Analysis D).

*N* (individuals)	247,848
Gender	
Female	153,187 (61.8%)
Male	94,661 (38.2%)
Age (years)	51.8 ± 15.1
Age category	
30–39	63,929 (25.79%)
40–49	53,888 (21.74%)
50–59	55,087 (22.23%)
60–69	40,426 (16.31%)
70–79	22,183 (8.95%)
80–89	10,578 (4.27%)
≥ 90	1757 (0.71%)
Weight (kg)	76.8 ± 17.4
Height (cm)	166 ± 10
BMI (kg/m^2^)	28.3 ± 5.8
< 18.5 Underweight	2832 (1.60%)
18.5–24.9 Normal	49,649 (28.11%)
25–29.9 Overweight	63,692 (36.06%)
≥ 30 Obese	60,474 (34.23%)
BP systolic (mmHg)	126 ± 18
BP diastolic (mmHg)	76.1 ± 10.2
BP category	
Hypertension 1	53,456 (21.8%)
Hypertension 2	44,580 (18.2%)
Normal BP	146,660 (59.9%)
Smoking status	
Non‐smoker	184,003 (78.46%)
Past smoker	5680 (2.42%)
Smoker	44,822 (19.11%)
~missing~	13,343 (5.38%)
Ethnic group	
Arab	47,015 (19.0%)
General	164,408 (66.3%)
Jewish Ultra‐orthodox	36,425 (14.7%)

*Note:* Characteristics of 247,848 LHS members aged ≥ 30 years included in the longitudinal supplementation analysis (Analysis D). This cohort includes all individuals with at least one serum 25(OH)D measurement during the study window and available baseline 25(OH)D levels after excluding those for whom no test could be found while not under supplementation (> 0.2 ng/mL predicted contribution). Continuous variables are presented as mean ± SD, and categorical variables as counts (percentages).

**FIGURE 5:**
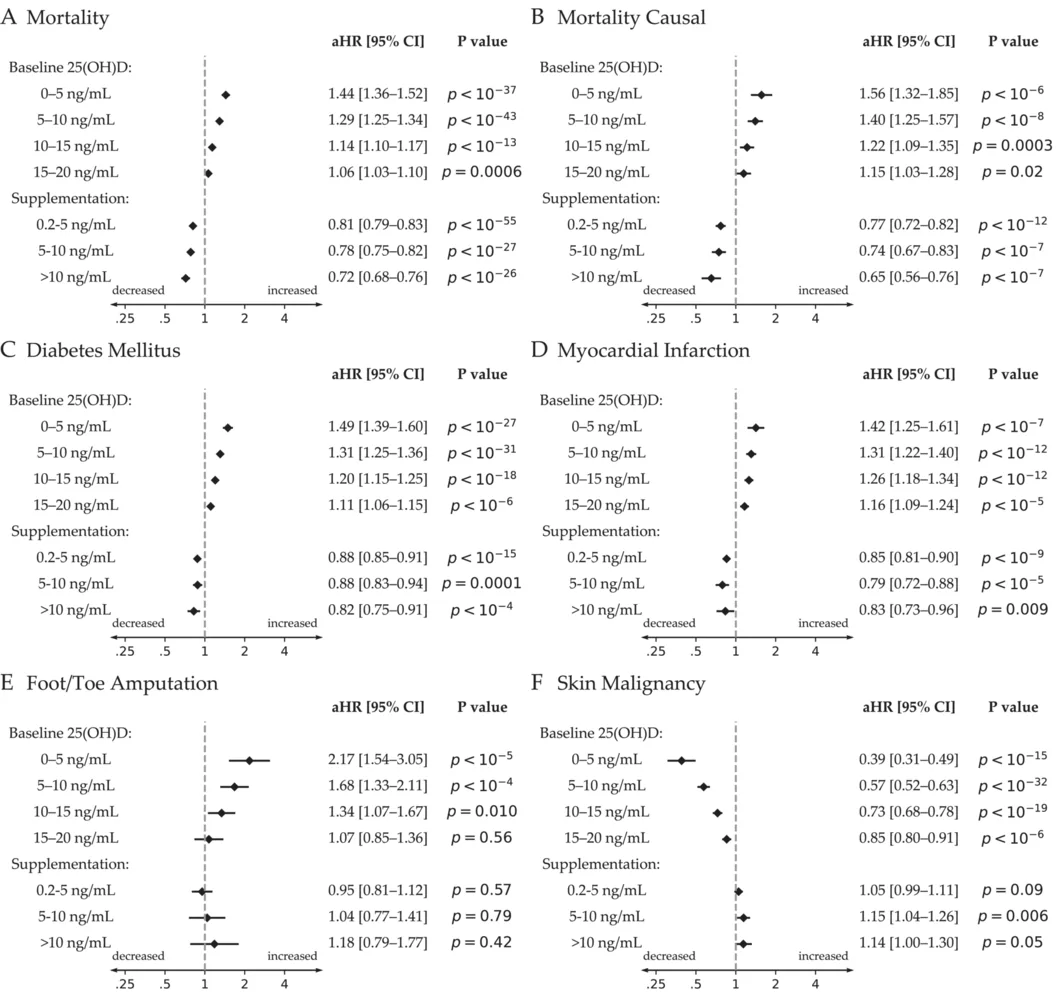
Longitudinal joint effects of baseline vitamin D status and supplementation on health outcomes in LHS (Analysis D). Adjusted hazard ratios (HRs) with 95% confidence intervals (log scale) are shown for mortality and four clinical outcomes among all Leumit Health Services (LHS) members aged ≥ 30 years who had at least one serum 25(OH)D measurement since 2009. The index date was defined as the first available measurement within the study window. Cox proportional‐hazards models jointly included baseline serum 25(OH)D categories (0–5, 5–10, 10–15, 15–20 ng/mL; reference > 20 ng/mL) and vitamin D supplementation categories modeled time‐dependently from pharmacy dispensing records (0.2–5, 5–10, > 10 ng/mL serum‐equivalent; reference < 0.2 ng/mL). Models were adjusted for age, sex, smoking status, socioeconomic status, BMI (fine categories plus offset), estimated GFR category, albuminuria category, systolic blood pressure, hypertension, and baseline diabetes, and applied a 6‐month lag to minimize reverse causation from early events. Panel B presents results from inverse‐probability of treatment stabilized weighting (IPTW) marginal structural Cox models for mortality, using cumulative stabilized weights to account for time‐varying confounding of supplementation by prior measured covariates. Stabilized weights were truncated at the 1st and 99th percentiles to reduce the influence of extreme values. This causal‐framework sensitivity analysis was performed for all‐cause mortality, the primary endpoint. An HR < 1 indicates lower risk; HR > 1 indicates higher risk. The vertical dashed line marks HR = 1. Panels: (A) Mortality (standard adjusted model), (B) Mortality (IPTW marginal structural model), (C) Diabetes mellitus, (D) Myocardial infarction, (E) Foot/Toe amputation, (F) Skin malignancy. Higher baseline 25(OH)D and increasing supplementation dose were consistently associated with lower risks of outcomes in panels A‐D. Panel F served as a negative‐control outcome: It showed an inverse association with baseline vitamin D, likely reflecting higher sun exposure among individuals with higher baseline 25(OH)D, but no consistent risk association with supplementation calculated from pharmacy dispensing data.

For mortality (Figure 5A), baseline vitamin D deficiency was associated with a graded, dose–dependent increase in risk: HR = 1.44 [1.36–1.52] for < 5 ng/mL; HR = 1.29 [1.25–1.34] for 5–10 ng/mL; HR = 1.14 [1.10–1.17] for 10–15 ng/mL; and HR = 1.06 [1.03–1.10] for 15–20 ng/mL (reference > 20 ng/mL; all *p* < 0.001). Vitamin D supplementation was independently associated with lower risk in a dose–dependent fashion: HR = 0.72 [0.68–0.76] for > 10 ng/mL serum–equivalent; HR = 0.78 [0.75–0.82] for 5–10 ng/mL; and HR = 0.81 [0.79–0.83] for 0.2–5 ng/mL (all *p* < 10^−26^).

To strengthen causal interpretation, we additionally performed inverse–probability of treatment weighted (IPTW) marginal structural Cox analyses for mortality (Figure 5B). In these causal–framework analyses, the graded association between baseline vitamin D deficiency and mortality remained evident and became slightly stronger: HR = 1.56 [1.32–1.85] for < 5 ng/mL; HR = 1.40 [1.25–1.57] for 5–10 ng/mL; HR = 1.22 [1.09–1.35] for 10–15 ng/mL; and HR = 1.15 [1.03–1.28] for 15–20 ng/mL. Supplementation also remained independently protective in a dose_−_dependent manner, with HR = 0.65 [0.56–0.76] for > 10 ng/mL serum–equivalent; HR = 0.74 [0.67–0.83] for 5–10 ng/mL; and HR = 0.77 [0.72–0.82] for 0.2–5 ng/mL (all *p* < 10^−7^).

For diabetes mellitus (Figure 5C), lower baseline vitamin D was strongly associated with higher risk: HR = 1.49 [1.39–1.60] for < 5 ng/mL; HR = 1.31 [1.25–1.36] for 5–10 ng/mL; HR = 1.20 [1.15–1.25] for 10–15 ng/mL; and HR = 1.11 [1.06–1.15] for 15–20 ng/mL (all *p* < 10^−6^).

Supplementation was again associated with lower risk: HR = 0.88 [0.85–0.91] for 0.2–5 ng/mL; HR = 0.88 [0.83–0.94] for 5–10 ng/mL; and HR = 0.82 [0.75–0.91] for > 10 ng/mL (all *p* < 0.001).

For myocardial infarction (Figure 5D), low baseline vitamin D was linked to higher risk (HR = 1.42 [1.25–1.61] for < 5 ng/mL; HR = 1.31 [1.22–1.40] for 5–10 ng/mL; HR = 1.26 [1.18–1.34] for 10–15 ng/mL; HR = 1.16 [1.09–1.24] for 15–20 ng/mL; all *p* < 10^−5^), and supplementation again showed significantly decreased risk, including HR = 0.85 [0.81–0.90] for 0.2–5 ng/mL, HR = 0.79 [0.72–0.88] for 5–10 ng/mL, and HR = 0.83 [0.73–0.96] for > 10 ng/mL.

For foot/toe amputation (Figure 5E), a strong baseline dose–response was evident, with HR = 2.17 [1.54–3.05] for < 5 ng/mL; HR = 1.68 [1.33–2.11] for 5–10 ng/mL; and HR = 1.34 [1.07–1.67] for 10–15 ng/mL. No significant inverse association with supplementation was detected, likely due to the limited number of amputation events under supplementation.

For skin malignancy (Figure 5F), baseline vitamin D showed an inverse association, whereas supplementation showed no corresponding association. In subtype analyses, severe baseline deficiency remained inversely associated with non‐melanoma skin cancer across 6‐, 12‐, and 24‐month landmark lags, while the LHS melanoma estimates were inverse but less precise. In the time‐dependent supplementation analyses, supplementation showed no significant association with melanoma, while hazard ratios for non‐melanoma skin cancer were modestly increased (Table S7).

These findings demonstrate that lower baseline vitamin D levels are associated with increased risks across multiple aging‐related outcomes, while supplementation, modeled independently of baseline status, was associated with dose‐dependent reductions in mortality, metabolic disease, and cardiovascular risk. Importantly, the mortality association remained robust in causal‐framework sensitivity analyses using inverse‐probability weighted marginal structural Cox models, which were specifically designed to reduce bias from time‐varying treatment selection and measured confounding. Similarly, the associations between time‐dependent supplementation and mortality, diabetes mellitus, and myocardial infarction remained materially unchanged when the lag was extended from 6 months to 12 months and 24 months (Table S6).

### 3.7 Risk Reduction and Number Needed to Treat

To evaluate the clinical utility of vitamin D supplementation, we estimated the 10‐year number needed to treat (NNT) to prevent one all‐cause mortality event, stratified by age group (≥ 30, ≥ 50, and ≥ 70 years), baseline serum 25(OH)D category, and the predicted supplementation‐related increase in serum levels (Figure 6). Because mortality represents the only outcome not subject to potential competing‐risk bias, NNT estimation was restricted to all‐cause mortality.

**FIGURE 6:**
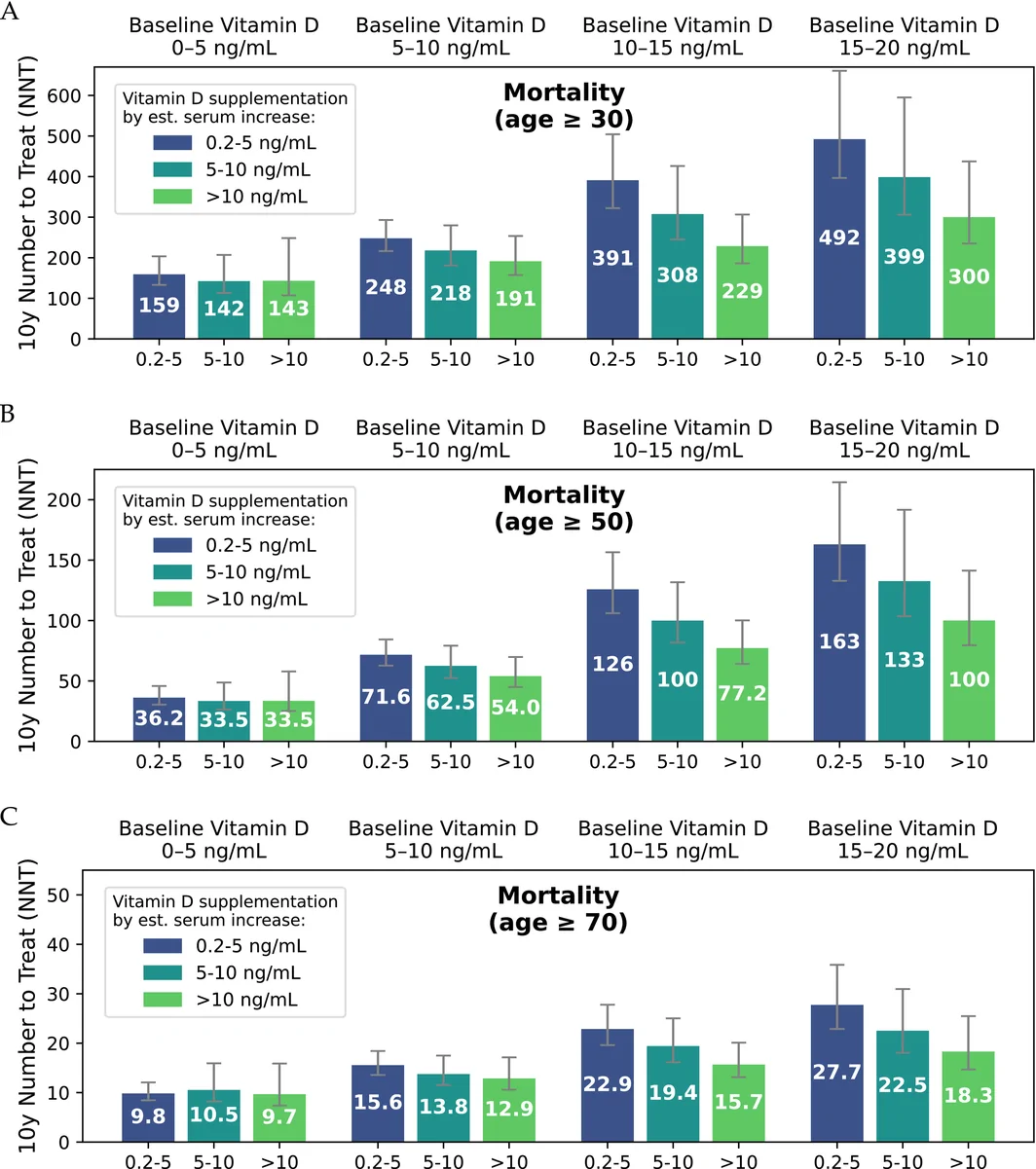
Estimated 10‐year number needed to treat (NNT) for prevention of all‐cause mortality by age, baseline vitamin D status, and supplementation level in LHS (Analysis D). Panels A–C show the estimated 10‐year number needed to treat (NNT) to prevent one death for each vitamin D supplementation category (color‐coded bars), stratified by baseline serum 25(OH)D concentration (groups of columns) and by age threshold (rows: ≥ 30, ≥ 50, ≥ 70 years). Supplementation categories are defined by the modeled increase in serum 25(OH)D (ng/mL). Error bars represent 95% confidence intervals. NNT values were derived from absolute risk reductions estimated from the joint time‐dependent Cox proportional‐hazards models (see Methods). NNT declined markedly with older age and with lower baseline 25(OH)D (< 10 ng/mL), reaching approximately 10–15 in older adults with very low baseline levels, corresponding to substantial estimated absolute risk reductions in these high‐risk groups.

Estimated mortality risk reductions were evident at all ages and for all baseline vitamin D categories below 20 ng/mL, with NNTs declining sharply with advancing age. Among participants with baseline 25(OH)D < 5 ng/mL, NNTs among participants aged ≥ 30 ranged from 142 to 159 depending on supplementation dose, but improved markedly to 33.5–36.2 among participants aged ≥ 50 and as low as 9.7–10.5 among participants aged ≥ 70.

For those starting at 5–10 ng/mL, NNTs similarly decreased from 191–248 at age 30 to 54.0–71.6 at age 50 and 12.9–15.6 at age 70. At baseline levels of 10–15 ng/mL, NNTs dropped from 229–391 at age 30 to 77.2–126 at age 50 and 15.7–22.9 at age 70. Even among individuals with baseline 15–20 ng/mL, NNTs remained clinically meaningful in older adults, declining from 300–492 at age 30 to 100–163 at age 50 and 18.3–27.7 at age 70.

## 4 Discussion

In this dual‐cohort longitudinal analysis leveraging real‐world data from two countries, severe vitamin D deficiency consistently identified a systemic state of increased aging‐related vulnerability spanning metabolic, vascular, renal, neurodegenerative, and mortality‐related domains. Importantly, our analyses suggest that vitamin D supplementation, modeled through pharmacy records and validated serum responses, is associated with significant reductions in risk across a wide range of clinical outcomes. These reductions were consistent, dose‐dependent, and remained significant even after controlling for confounding demographic, behavioral, and socioeconomic factors. Notably, the observed associations extended across multiple major age‐related outcomes and organ systems, supporting the concept that vitamin D status may function as a broad determinant of systemic aging vulnerability and multimorbidity accumulation rather than merely a disease‐specific biomarker.

The LHS matched cohort design allowed for tight baseline control of demographic and clinical risk factors, including BMI, blood pressure, diabetes, smoking status, and kidney disease. Findings were replicated in an independent, US‐based federated health network (TriNetX), enhancing external validity. Age at index and current age at data extraction were closely aligned between the matched groups, indicating similar calendar timing of the index measurements and reducing the likelihood that secular changes in vitamin D status reported in population studies (Pfeiffer et al. 2026) account for the observed between‐group differences.

A major strength of this study is the integration of detailed laboratory, demographic, and pharmacy data from a national health organization, enabling precise classification of baseline vitamin D status and estimation of serum responses to supplementation. We leveraged these data to construct a time‐dependent covariate for vitamin D supplementation, derived from pharmacy‐dispensed records and calibrated to predict serum 25(OH)D changes over time. This approach helped distinguish the potentially modifiable effect of supplementation from the confounding influence of demographic, socioeconomic, and obesity‐related determinants of vitamin D deficiency. Importantly, supplementation exposure was modeled longitudinally throughout follow‐up, enabling assessment of dynamic exposure‐response relationships rather than reliance on baseline supplementation status alone. The supplementation associations remained directionally consistent in the IPTW marginal structural mortality analysis. The absence of a corresponding risk association for melanoma or a risk‐reduction pattern for non‐melanoma skin cancer (Table S7), despite strong inverse associations with baseline vitamin D status, further argues against healthy‐user bias or ultraviolet‐exposure confounding as likely explanations. A generalized healthy‐user effect would be expected to produce lower risks across outcomes, whereas confounding by ultraviolet exposure would be expected to operate in the opposite direction for skin cancer. The absence of either coherent pattern in the supplementation analyses argues against these mechanisms as primary explanations for the observed associations with mortality and other aging‐related outcomes.

Our findings revealed strikingly low numbers needed to treat (NNTs) for all‐cause mortality. Notably, the estimated NNTs in older severely deficient adults fall within ranges considered clinically meaningful for established preventive interventions. Even moderate supplementation (predicted serum increase of 10–20 ng/mL) in this group yielded substantial absolute benefit. Importantly, benefit extended beyond the severely deficient: individuals with baseline 25(OH)D of 15–20 ng/mL also showed meaningful reductions in mortality risk, with NNTs of roughly 18–28 depending on supplementation level. These results highlight vitamin D supplementation as a scalable, low‐cost intervention with significant public‐health potential when targeted to individuals with suboptimal baseline levels. From a geroscience perspective, these findings are notable because interventions capable of simultaneously influencing mortality, cardiometabolic disease, vascular complications, and functional decline remain relatively uncommon in population‐scale human datasets.

Previous randomized controlled trials (RCTs) of vitamin D supplementation have yielded inconsistent results for chronic disease and mortality outcomes (Chen et al. 2024; Davidson et al. 2013; Zhang et al. 2019; Zittermann et al. 2017). Several factors likely explain this discrepancy. Most RCTs enrolled participants regardless of baseline vitamin D status, resulting in limited power to detect benefits confined to the severely deficient subgroups, where our strongest associations were observed, particularly among older adults. In addition, background supplement use in placebo groups and incomplete adherence may dilute between‐group differences in achieved 25(OH)D levels, leading to underestimation of true effects. Moreover, many trials used fixed low doses, without achieving the serum repletion levels associated with reduced mortality and morbidity in our real‐world data. Consistent with the importance of achieved exposure, a secondary diabetes analysis found progressively lower diabetes risk with higher intratrial 25(OH)D concentrations among participants assigned to vitamin D (Dawson‐Hughes et al. 2020). These limitations help reconcile the apparent divergence between large pragmatic RCTs and the consistent, dose‐dependent associations observed in our cohorts.

The biological mechanisms underlying these protective effects remain to be fully elucidated. One plausible explanation is that vitamin D status influences shared upstream aging‐related pathways, potentially including chronic inflammation, immune dysregulation, endothelial dysfunction, microbial‐host interactions, and impaired tissue resilience (Israel et al. 2022; Lagishetty et al. 2011; Merzon et al. 2020; Schwalfenberg 2011; Singh et al. 2020). The breadth of associated outcomes observed across cardiovascular, metabolic, renal, neurodegenerative, and mortality‐related domains is compatible with mechanisms operating at the level of systemic organismal resilience rather than isolated disease‐specific pathways. However, the precise biological mechanisms underlying these associations remain incompletely understood and require further mechanistic investigation. Supporting this hypothesis is the breadth of conditions impacted—including diabetes, cardiovascular disease, and all‐cause mortality—which may share common microbial or immune‐mediated origins. Within this framework, we recently proposed that persistent *Toxoplasma gondii* infection may contribute to late‐life morbidity through delayed pathogenicity, and that vitamin D deficiency could facilitate this process by creating a less effective immune environment for containment of latent intracellular infection (Israel and Israel 2026).

A particularly notable observation was the strong association between severe vitamin D deficiency and subsequent foot/toe amputation. Given that such amputations are frequently precipitated by persistent infection, this finding further supports a microbiologically mediated mechanism (Joshi et al. 2023). Our findings raise the possibility that severe vitamin D deficiency may contribute to the elevated burden of lower‐extremity complications observed in certain high‐risk population subgroups.

This study has limitations. As an observational analysis, residual confounding cannot be entirely excluded despite extensive matching, adjustment, and validation across two independent cohorts. Vitamin D supplementation may also co‐occur with other unmeasured health‐promoting behaviors. In addition, supplementation exposure was derived from pharmacy‐dispensed vitamin D products within LHS‐affiliated pharmacies; dispensing does not ensure ingestion, and over‐the‐counter purchases through untracked channels may not have been captured.

Nevertheless, the strength, consistency, and dose‐dependent pattern of associations across cohorts support a genuine underlying relationship. Importantly, the persistence of the mortality associations within IPTW marginal structural models suggests that the observed protective effects are unlikely to be explained solely by measured treatment‐selection bias or time‐varying confounding. Although standard Cox models treat death as a censoring event, mortality itself functions as a competing outcome for nonfatal events. Finally, although causal inference from observational data necessarily depends on assumptions of exchangeability and adequate capture of confounders, the convergence of findings across conventional multivariable models, longitudinal time‐dependent supplementation analyses, dose–response relationships, and IPTW causal‐framework analyses substantially strengthens the plausibility of a modifiable biological effect.

In conclusion, our findings support severe vitamin D deficiency as a prevalent and potentially modifiable determinant of aging‐related vulnerability across multiple organ systems. The consistency of the associations across independent cohorts, longitudinal exposure modeling, dose–response relationships, and causal‐framework sensitivity analyses supports the plausibility that vitamin D status may influence systemic resilience and multimorbidity trajectories during aging. These findings provide a population‐scale framework for future mechanistic and interventional studies investigating vitamin D biology within the context of human aging.

## Author Contributions

Conceptualization: Ariel Israel, Eugene Merzon, Eli Magen. Methodology: Ariel Israel, Eugene Merzon. Investigation: Ariel Israel, Sarah Israel, Eugene Merzon. Writing – original draft: Ariel Israel. Writing – review and editing: Ariel Israel, Abraham Weizman, Sarah Israel, Shai Ashkenazi, Shlomo Vinker, Eli Magen, Eugene Merzon, Eytan Ruppin.

## Funding

This study was funded internally by Leumit Health Services.

## Disclosure

*Materials & Correspondence*: Correspondence and requests for materials should be addressed to the corresponding author.

## Conflicts of Interest

The authors declare no conflicts of interest.

## Supporting information


**Figure S1:** Flowchart of Matched Vitamin D Cohort Construction and Matching.
**Figure S2:** Sensitivity analyses performed with a 12‐month lag.
**Table S1:** Seasonal Regression Model of Log‐Transformed Serum Vitamin D Levels.
**Table S2:** Multivariable OLS Regression Predicting Log‐Transformed Serum Vitamin D Levels.
**Table S3:** Regression model for vitamin D supplementation.
**Table S4:** Predefined medical‐condition categories screened in LHS and ICD‐9‐CM definitions of reported outcomes.
**Table S5:** Twenty‐four‐month landmark sensitivity analyses for severe vitamin D deficiency versus sufficiency in LHS and TriNetX.
**Table S6:** LHS time‐dependent vitamin D supplementation associations under 6‐, 12‐, and 24‐month landmark lags.
**Table S7:** Melanoma and non‐melanoma skin‐cancer subtype analyses in LHS and TriNetX.
