# Supplementary material for: Vitamin D Deficiency, Supplementation, and Aging‐Related Morbidity and Mortality Across Two Longitudinal Population Cohorts
Source: Aging Cell. 2026 Oct 8;25(10):e70752. doi: 10.1111/acel.70752 (PMC13650182; doi:10.1111/acel.70752)
Supplement: Supplementary file 1 [file ACEL-25-e70752-s001.docx]

**Supplementary Material for**

**Vitamin D Deficiency, Supplementation, and Aging-Related Morbidity and Mortality Across Two Longitudinal Population Cohorts**

Ariel Israel^1,2^*, Abraham Weizman^3^, Sarah Israel^4^,
Shai Ashkenazi^5,6^, Eytan Ruppin^7^, Eli Magen^8^, Eugene Merzon^1,5^, Shlomo Vinker^1^

Contents

[Supplementary Methods 1 - Construction and calibration of the longitudinal vitamin D supplementation exposure	2](#_Toc240992062)

[Supplementary Methods 2 - Time-dependent outcome models, IPTW marginal structural analysis, absolute-risk estimation, and lag sensitivity analyses	3](#_Toc240992063)

[Supplementary Table 1. Seasonal Regression Model of Log-Transformed Serum Vitamin D Levels	5](#_Toc240992064)

[Supplementary Table 2. Multivariable OLS Regression Predicting Log-Transformed Serum Vitamin D Levels	6](#_Toc240992065)

[Supplementary Table 3. Regression model for vitamin D supplementation	7](#_Toc240992066)

[Supplementary Table 4. Predefined medical-condition categories screened in LHS and ICD-9-CM definitions of reported outcomes	8](#_Toc240992067)

[Supplementary Table 5. Twenty-four-month landmark sensitivity analyses for severe vitamin D deficiency versus sufficiency in LHS and TriNetX	11](#_Toc240992068)

[Supplementary Table 6. LHS time-dependent vitamin D supplementation associations under 6-, 12-, and 24-month landmark lags	12](#_Toc240992069)

[Supplementary Table 7. Melanoma and non-melanoma skin-cancer subtype analyses in LHS and TriNetX	13](#_Toc240992070)

[Supplementary Figure 1. Flowchart of Matched Vitamin D Cohort Construction and Matching	14](#_Toc240992071)

[Supplementary Figure 2. Sensitivity analyses performed with a 12-month lag	15](#_Toc240992072)

## Supplementary Methods 1 - Construction and calibration of the longitudinal vitamin D supplementation exposure

All vitamin D-containing products dispensed through Leumit Health Services (LHS) pharmacies were extracted from pharmacy records. Each product was assigned a total vitamin D content, expressed in kilo-international units (kIU), based on the labeled concentration and number of dosage units contained in the dispensed package.

To estimate the contribution of documented supplementation to serum 25-hydroxyvitamin D [25(OH)D] concentrations, we used a two-stage regression framework. In the first stage, the natural logarithm of measured serum 25(OH)D was modeled using demographic, anthropometric, socioeconomic, calendar-time, and seasonal variables. Predictors included sex/ethnic group, socioeconomic-status categories, detailed BMI categories and within-category BMI offsets, age, calendar year, and first- and second-order annual sine and cosine terms. This baseline model explained 25.4% of the variation in log-transformed serum 25(OH)D (Supplementary Table 2).

In the second stage, residual variation in 25(OH)D unexplained by the baseline model was regressed on preceding vitamin D purchases. Supplementation was represented by cumulative dispensed vitamin D amounts over monthly, quarterly, and yearly exposure intervals. The fitted coefficients for these exposure windows are provided in Supplementary Table 3. Recent purchases contributed most strongly to measured serum 25(OH)D, with progressively smaller coefficients for more remote exposure periods.

Clinical variables were not entered into this second-stage supplementation model, in order to avoid allowing clinical status potentially affected by vitamin D deficiency or supplementation to determine the estimated supplementation exposure. The resulting coefficients were used to calculate, for each individual and follow-up interval, the predicted serum 25(OH)D contribution attributable to documented supplementation.

Baseline 25(OH)D was classified as 0–5, 5–10, 10–15, 15–20, or >20 ng/mL, with >20 ng/mL serving as the reference category. The time-updated predicted supplementation contribution was categorized as 0–0.2 ng/mL-equivalent (reference), 0.2–5 ng/mL, 5–10 ng/mL, or >10 ng/mL.

For longitudinal outcome analyses, person-time was split into monthly intervals and supplementation exposure was updated over follow-up. Cox proportional-hazards models jointly included baseline 25(OH)D category and the time-dependent supplementation category and were adjusted for age, sex, smoking status, socioeconomic status, BMI category and within-category offset, systolic blood pressure, hypertension, eGFR category, albuminuria category, and baseline diabetes.

## Supplementary Methods 2 - Time-dependent outcome models, IPTW marginal structural analysis, absolute-risk estimation, and lag sensitivity analyses

**Time-dependent Cox models**

Follow-up was represented by individual-month intervals, with the supplementation exposure updated according to pharmacy-dispensing history. Conventional Cox models jointly incorporated baseline 25(OH)D status and time-updated supplementation exposure. Robustness to early reverse causation was evaluated using progressively longer post-index landmark lags as described below.

**IPTW marginal structural mortality analysis**

To address measured treatment-selection processes in the longitudinal supplementation analysis, a causal-framework sensitivity analysis was performed for all-cause mortality using inverse-probability-of-treatment-weighted (IPTW) marginal structural Cox models.

Stabilized treatment weights were estimated for each individual-month interval using multinomial logistic regression models for the observed time-updated supplementation category. The numerator model estimated the probability of the observed supplementation category conditional on prior supplementation history. The denominator model additionally incorporated measured covariates including baseline 25(OH)D category, age, sex, smoking status, socioeconomic status, BMI category and within-category BMI offset, baseline diabetes, chronic kidney disease, hypertension, systolic blood pressure, eGFR category, and albuminuria category.

Interval-specific stabilized weights were accumulated longitudinally to obtain individual treatment weights. To limit the influence of extreme observations, stabilized weights were truncated at the 1st and 99th percentiles. Weighted Cox proportional-hazards models were then fitted using robust sandwich variance estimates clustered by individual. The IPTW analysis was restricted to all-cause mortality, the primary endpoint.

**Absolute risk reduction and number needed to treat**

Ten-year absolute risk reductions (ARRs) and corresponding numbers needed to treat (NNTs) were derived from the joint time-dependent Cox models. Analyses were stratified by baseline 25(OH)D category and age threshold (≥30, ≥50, and ≥70 years).

For each baseline stratum, a standardized reference profile was defined using mean continuous covariates and modal categorical covariates among individuals in the reference supplementation category (<0.2 ng/mL-equivalent). Adjusted survival probabilities were then projected under each supplementation category. At time $t$, the absolute risk reduction was calculated from the difference in projected survival between the supplementation and reference conditions:

ARR(t) = S₁(t) − S₀(t)

NNT(t) = 1 / |ARR(t)|.

Ten-year estimates are presented in Figure 6.

**Landmark-lag sensitivity analyses**

The primary longitudinal analyses used a 6-month lag after the qualifying serum 25(OH)D measurement to reduce the influence of pre-existing or incipient disease. Sensitivity analyses were repeated using 12-month and 24-month landmark lags while retaining the same exposure definitions, covariate adjustment, and outcome definitions.

These analyses were performed for the LHS matched severe-deficiency versus sufficiency cohort, the corresponding TriNetX validation analyses, and the principal LHS time-dependent supplementation analyses. The 24-month matched-cohort sensitivity estimates are reported in Supplementary Table 5; 6-, 12-, and 24-month supplementation estimates are reported in Supplementary Table 6; and the corresponding skin-cancer subtype analyses are reported in Supplementary Table 7

## Supplementary Table 1. Seasonal Regression Model of Log-Transformed Serum Vitamin D Levels

| **Variable** | **Coefficient (β)** | **Std. Error** | **t-Statistic** | **P-value** | **95% CI Lower** | **95% CI Upper** |
| --- | --- | --- | --- | --- | --- | --- |
| Intercept | 2.8958 | 0.0005 | 5626.8609 | <0.0001 | 2.8948 | 2.8968 |
| season_sin | -0.1008 | 0.0007 | -135.9558 | <0.0001 | -0.1022 | -0.0993 |
| season_cos | -0.1088 | 0.0007 | -152.4612 | <0.0001 | -0.1102 | -0.1074 |
| season_sin² | -0.0160 | 0.0007 | -22.5060 | <0.0001 | -0.0174 | -0.0146 |
| season_cos² | 0.0068 | 0.0007 | 9.1851 | <0.0001 | 0.0054 | 0.0083 |

**Model Summary:**

- Dependent variable: log_vitamin_d
- R-squared = 0.040
- Adjusted R-squared = 0.040
- F-statistic = 10,473 (p < 0.0001)

This regression model estimates the seasonal variation in serum 25(OH)D levels using sinusoidal and second-harmonic terms (sine and cosine). The outcome variable was the natural logarithm of vitamin D levels. Seasonal terms are based on calendar date and are independent of individual-level characteristics. The analysis includes over 1.65 million observations from Leumit Health Services (LHS). The model explains a modest portion of variance (R^2^ = 0.040), reflecting significant impact of seasonality, but also high interindividual variability in serum 25(OH)D.

## Supplementary Table 2. Multivariable OLS Regression Predicting Log-Transformed Serum Vitamin D Levels

| **Variable** | **Coefficient** | **Std. Error** | **t-Statistic** | **P-value** | **95% CI Lower** | **95% CI Upper** |
| --- | --- | --- | --- | --- | --- | --- |
| Intercept | 2.7542 | 0.0033 | 834.3379 | <0.0001 | 2.7477 | 2.7606 |
| Female, Arab | -0.5713 | 0.0020 | -291.9690 | <0.0001 | -0.5752 | -0.5675 |
| Female, Ultra-Orthodox | -0.1533 | 0.0019 | -79.4336 | <0.0001 | -0.1571 | -0.1495 |
| Male, General | 0.0181 | 0.0014 | 12.8078 | <0.0001 | 0.0153 | 0.0209 |
| Male, Arab | -0.1802 | 0.0026 | -70.6107 | <0.0001 | -0.1852 | -0.1752 |
| Male, Ultra-Orthodox | -0.1031 | 0.0025 | -40.6715 | <0.0001 | -0.1081 | -0.0982 |
| SES 4–6 | 0.0788 | 0.0025 | 32.0119 | <0.0001 | 0.0739 | 0.0836 |
| SES 7–9 | 0.0841 | 0.0026 | 32.3250 | <0.0001 | 0.0790 | 0.0892 |
| SES 10–11 | 0.1302 | 0.0027 | 47.7027 | <0.0001 | 0.1249 | 0.1356 |
| SES 12–14 | 0.1316 | 0.0028 | 46.5542 | <0.0001 | 0.1260 | 0.1371 |
| SES ≥15 | 0.1437 | 0.0032 | 44.6216 | <0.0001 | 0.1374 | 0.1500 |
| SES Missing | 0.1180 | 0.0031 | 38.3047 | <0.0001 | 0.1119 | 0.1240 |
| BMI <18.5 (Underweight) | -0.0321 | 0.0048 | -6.6179 | <0.0001 | -0.0416 | -0.0226 |
| BMI 25–29.9 (Overweight) | -0.0657 | 0.0013 | -48.7713 | <0.0001 | -0.0684 | -0.0631 |
| BMI 30–34.9 (Obese I) | -0.1231 | 0.0016 | -76.8983 | <0.0001 | -0.1262 | -0.1199 |
| BMI 35–39.9 (Obese II) | -0.1794 | 0.0022 | -81.6797 | <0.0001 | -0.1837 | -0.1751 |
| BMI ≥40 (Obese III) | -0.2221 | 0.0028 | -79.4461 | <0.0001 | -0.2276 | -0.2166 |
| BMI per unit above boundary | -0.0086 | 0.0003 | -28.5125 | <0.0001 | -0.0092 | -0.0080 |
| BMI per unit below 18.5 | -0.0199 | 0.0031 | -6.4609 | <0.0001 | -0.0260 | -0.0139 |
| Years since 2009 | 0.0173 | 0.0002 | 95.2821 | <0.0001 | 0.0169 | 0.0176 |
| Age | 0.0026 | 0.0000 | 82.2403 | <0.0001 | 0.0025 | 0.0027 |
| season_sin | -0.1151 | 0.0007 | -155.7826 | <0.0001 | -0.1165 | -0.1136 |
| season_cos | -0.1052 | 0.0007 | -149.7457 | <0.0001 | -0.1066 | -0.1039 |
| season_sin² | -0.0147 | 0.0007 | -20.9502 | <0.0001 | -0.0161 | -0.0133 |
| season_cos² | 0.0009 | 0.0007 | 1.2735 | 0.2028 | -0.0005 | 0.0024 |

**Model Summary:**

• **Dependent variable:** log_vitamin_d

• **R-squared:** 0.254

• **Adjusted R-squared:** 0.254

• **F-statistic:** 11,301 (p < 0.0001)

This table reports the results of a multivariable OLS regression model predicting serum 25(OH)D levels in adult LHS members with available demographic, socioeconomic, and BMI data. Independent variables include sex, ethnicity, socioeconomic status (SES), detailed BMI categories, age, year of test, and seasonal sinusoidal terms to model calendar trends. Negative coefficients indicate lower vitamin D levels associated with the corresponding category. The model explains 25.4% of the variance in log-transformed vitamin D levels.

## Supplementary Table 3. Regression model for vitamin D supplementation

| **Variable** | **Coefficient** | **Std. Error** | **t-Statistic** | **P-value** | **95% CI Lower** | **95% CI Upper** |
| --- | --- | --- | --- | --- | --- | --- |
| **vitd_kiu_m0** | 0.0127 | 0.0003 | 43.9128 | <0.0001 | 0.0121 | 0.0132 |
| **vitd_kiu_m1** | 0.0217 | 0.0003 | 68.0262 | <0.0001 | 0.0211 | 0.0223 |
| **vitd_kiu_m2** | 0.0216 | 0.0003 | 68.7724 | <0.0001 | 0.0210 | 0.0222 |
| **vitd_kiu_m3** | 0.0195 | 0.0003 | 64.0880 | <0.0001 | 0.0189 | 0.0201 |
| **vitd_kiu_m4** | 0.0148 | 0.0003 | 46.8482 | <0.0001 | 0.0142 | 0.0154 |
| **vitd_kiu_q1** | 0.0121 | 0.0003 | 39.3332 | <0.0001 | 0.0115 | 0.0127 |
| **vitd_kiu_q2** | 0.0094 | 0.0002 | 52.0134 | <0.0001 | 0.0091 | 0.0098 |
| **vitd_kiu_q3** | 0.0057 | 0.0002 | 31.4716 | <0.0001 | 0.0054 | 0.0061 |
| **vitd_kiu_q4** | 0.0041 | 0.0002 | 22.4462 | <0.0001 | 0.0038 | 0.0045 |
| **vitd_kiu_q5** | 0.0038 | 0.0002 | 20.0053 | <0.0001 | 0.0035 | 0.0042 |
| **vitd_kiu_q6** | 0.0032 | 0.0002 | 16.0337 | <0.0001 | 0.0028 | 0.0036 |
| **vitd_kiu_q7** | 0.0021 | 0.0002 | 9.9826 | <0.0001 | 0.0017 | 0.0025 |
| **vitd_kiu_y2** | 0.0016 | 0.0001 | 18.3022 | <0.0001 | 0.0014 | 0.0018 |
| **vitd_kiu_y3** | 0.0006 | 0.0001 | 6.6200 | <0.0001 | 0.0005 | 0.0008 |
| **vitd_kiu_y4** | 0.0007 | 0.0001 | 7.3420 | <0.0001 | 0.0005 | 0.0009 |

This table presents the results of a robust linear regression model estimating the difference between observed and predicted serum vitamin D levels as a function of vitamin D supplementation intensity over time. The dependent variable is the difference between measured serum vitamin D levels and the model-based estimate. Independent variables represent cumulative supplementation (in thousands of IU) over various time windows: monthly (vitd_kiu_m0 to m4), quarterly (q1 to q7), and yearly intervals (y2 to y4). All coefficients are statistically significant (p < 0.0001).

## Supplementary Table 4. Predefined medical-condition categories screened in LHS and ICD-9-CM definitions of reported outcomes

A. List of considered categories

ATRIAL FIBRILLATION

ATRIAL FLUTTER

ADHD

ALCOHOLISM

ALZHEIMER

ANKYLOSING SPONDYLITIS

AORT ANEURYSM

ASTHMA

AUTISM

BEHCET

CA BREAST

CA COLON

CA KIDNEY

CA LUNG

CA METASTATIC

CA PANCREAS

CA PROSTATE

CA SKIN

CA SKIN BASAL

CA SKIN SQUAMOUS

CABG

CARDIOMYOPATHY

CELIAC

CEREBRAL BLEED

CHF

CIRRHOSIS

CKD

COAGULATION DISORDER

CONGENITAL HEART

CONNECT TISSUE DIS

COPD

CROHN'S DISEASE

CVA

DEAFNESS

DEMENTIA

DEPRESSION

DERMATOMYOSITIS

DIABETES INSIPIDUS

DIABETIC RETINOPATHY

DIALYSIS

DM

DM EYE DAMAGE

DM NEUROPATHY

DOWN

DRUG

DVT

DYSLIP

EATING DISORDER

EMBOLISM

ENDOCARDITIS

EPILEPSY

FIBROMYALGIA

FMF

G6PD DEF

GI BLEEDING

GI MALIGNANCY

GOUT

GRAVES

HASHIMOTO

HEMATOLOGIC MALIGNANCY

HEMIPLEGIA

HEPATITIS A

HEPATITIS AUTOIMMUNE

HEPATITIS B

HEPATITIS C

HEPATOCELLULAR CARCINOMA

HIDRADENITIS SUPPURATIVA

HIP FRACT

HTN

HYPERPARATHYROIDISM

HYPOTHYROIDISM

IBD

IDIOPATHIC PULMONARY FIBROSIS

IHD

IMMUNOSUPPRESSION

LEG AMPUTATION

LIVER DIS

LIVER DIS SEVERE

MACE

MELANOMA

MI

MULTIPLE SCLEROSIS

MYOCARDITIS

OSA

OSTEOART

OSTEOPOROSIS

PARKINSON

PCI

PULMONARY EMBOLISM

PERICARDITIS

PERNICIOUS ANEMIA

POLYMYOSITIS

PSORIASIS

PSORIAT ARTHRITIS

PTSD

PEPTIC ULCER DISEASE

PULM CONGESTION

PULM HTN

PULMONARY HYPERTENSION

PVD

RHEUMATOID ARTHRITIS

SCHIZOPHRENIA

SCLERODERMA

SIADH

SJOGREN

SLE

SOLID TUMOR

THROMBOSIS

TIA

ULCERATIVE COLITIS

ULCERATIVE PROCTITIS

URINARY MALIGNANCY

VALVE DISEASE

VALVE REPAIR

VALVE REPLACEMENT

ABDOMINAL PAIN

ACNE

ALLERGIC CONJUNCTIVITIS

ALLERGIC RHINITIS

ALLERGIC URTICARIA

ALLERGY FOOD/UNSPECIFIED

ALOPECIA AREATA

ANXIETY

ATOPIC DERMATITIS

BACK PAIN

BRONCHIOLITIS

BRONCHITIS

CANDIDA

CELLULITIS

CHEST PAIN

CLUSTER HEADACHE

COGNITIVE DISORDER

CONSTIPATION

CONTACT DERMATITIS

COUGH

COVID

DERMATITIS HERPETIFORMIS

DERMATOPHYTOSIS

DIARRHEA

ECZEMA DYSHIDROTIC

ENTEROBIASIS

ERYTHEMA MULTIFORME

ERYTHEMA NODOSUM

FALL

FECAL INCONTINENCE

FINGER OR HAND INJURY

FLATULENCE

FRACTURE

GASTRITIS

GASTROENTERITIS

GERD

GINGIVITIS

H PYLORI

HEADACHE

HELMINTHIASIS

HEMOPTYSIS

HEMORRHOIDS

HERPES SIMPLEX

HERPES ZOSTER

HYPOGLYCEMIA

IDIOPATHIC URTICARIA

IMPETIGO

INCONTINENCE

INFLUENZA

INGUINAL HERNIA

INSOMNIA

ITP

KETOACIDOSIS

LICHEN PLANUS

LUPUS DISCOID

MOLLUSCUM CONTAGIOSUM

NASAL POLYP

NOCTURNAL ENURESIS

PANCREATITIS

PEDICULOSIS

PEMPHIGOID

PEMPHIGUS

PERSONALITY DISORDERS

PITYRIASIS ROSEA

PNEUMONIA BACTERIAL

PNEUMONIA UNSPECIFIED

POLYMYALGIA RHEUMATICA

RASH

ROSACEA

SARCOIDOSIS

SCABIES

SEBORRHEIC DERMATITIS

SINUSITIS

SOMATISATION

STAPH INFECTION

TINEA VERSICOLOR

TOE OR LEG INJURY

TONSILLITIS

TREMOR

URTI

UTI

VERTIGO/DIZINESS

VIRAL INFECTION

VIRAL WART

VITILIGO

WHEEZING/STRIDOR

AMBLYOPIA

AMD

ASTIGMATISM

BLEPHARITIS

BLINDNESS

CATARACT

CONJUNCTIVITIS

CONJUNCTIVITIS CHRONIC

CORNEAL DISORDERS

CORNEAL EROSION

DISORDERS OPTIC NERVE AND VISUAL PATHWAYS

DISORDERS VITREOUS BODY

DRY EYE

ENTROPION OR TRICHIASIS

EPIPHORA

EYELID INFLAMMATION

EYELID OTHER

GLAUCOMA

HORDEOLUM OR CHALZION

HYPERMETROPIA

KERATITIS

KERATOCONUS

MYOPIA

ORBIT DISORDERS

PERIORBITAL CELLULITIS

PTERYGIUM

PTOSIS

REFRACTION DISORDERS

RETINAL DETACHMENT

B. ICD-9 codes included in reported outcomes

| **Clinical variable** | **LHS category** | **ICD-9-CM codes (compact notation)** | **Role in analysis** |
| --- | --- | --- | --- |
| All-cause mortality | — | — | Mortality outcome; not diagnosis-code defined |
| Diabetes mellitus | DM | 250* | Outcome; diagnosis-based component of baseline diabetes covariate |
| Myocardial infarction | MI | 00.66; 36.01*; 36.02; 36.05; 36.06; 36.07; 36.1*; 410*; 411.0; 411.81; 412; 429.7*; V45.81; V45.82; V49.70 | Outcome |
| Cerebrovascular disease | CVA | 431; 434.01; 434.11; 434.9*; 436; 436.3 | Outcome |
| Dementia | DEMENTIA | 290*; 294.1*; 331.0; 331.1; 331.19; 331.82* | Outcome |
| Dialysis | DIALYSIS | V45.1*; V56* | Outcome |
| Diabetic retinopathy | DIABETIC RETINOPATHY | 250.5*; 362.0*; 362.29 | Outcome |
| Foot/toe amputation | LEG AMPUTATION | 84.1*; V49.7* | Outcome |
| Heart failure | CHF | 402.0*; 402.1*; 402.9*; 404.00; 416.11; 428* | Reported/screened outcome |
| Chronic kidney disease | CKD | 585; 585.2; 585.3; 585.4; 585.5; 585.6; 585.9 | Diagnosis-based component of baseline CKD covariate |
| Hypertension | HTN | 362.11; 401*; 402*; 403*; 404*; 405* | Diagnosis-based covariate |
| Skin malignancy | CA SKIN | 172*; 173* | Composite skin-cancer outcome |
| Melanoma | MELANOMA / CA SKIN subset | 172* | Skin-cancer subtype |
| Non-melanoma skin cancer | CA SKIN subset | 173* | Skin-cancer subtype |

**Note.** Panel B shows only diagnosis categories used in the reported outcomes or key diagnosis-based covariates; Panel A provides the complete exploratory screening library. Codes were taken directly from the supplied LHS diagnosis-category mapping (diag_categories.xlsx). A trailing asterisk denotes all mapped ICD-9-CM descendants of that stem present in the supplied category table; it does not imply additional unmapped codes. For melanoma/non-melanoma analyses, melanoma was defined by CA SKIN codes beginning 172 and non-melanoma skin cancer by CA SKIN codes beginning 173. Baseline CKD also incorporated reduced eGFR or severe albuminuria as specified in the main Methods. Only ICD-9-CM entries are displayed here.

## Supplementary Table 5. Twenty-four-month landmark sensitivity analyses for severe vitamin D deficiency versus sufficiency in LHS and TriNetX

| **Outcome** | **LHS adjusted HR (95% CI)** | **LHS P value** | **TriNetX HR (95% CI)** | **TriNetX P value** |
| --- | --- | --- | --- | --- |
| All-cause mortality | 2.39 (2.23–2.56) | 2.4×10^-134^ | 1.36 (1.33–1.40) | 1.4×10^-148^ |
| Diabetes mellitus | 1.26 (1.15–1.38) | 5.9×10^-7^ | 1.19 (1.16–1.22) | 1.7×10^-39^ |
| Myocardial infarction | 1.30 (1.13–1.49) | 1.7×10^-4^ | 1.21 (1.17–1.25) | 4.5×10^-25^ |
| Cerebrovascular disease | 1.18 (1.03–1.36) | 0.017 | 1.23 (1.18–1.27) | 2.6×10^-26^ |
| Dementia | 1.32 (1.19–1.47) | 3.2×10^-7^ | 1.08 (1.03–1.14) | 0.0015 |
| Dialysis | 3.84 (2.16–6.82) | 4.3×10^-6^ | 1.58 (1.49–1.68) | 1.1×10^-52^ |
| Diabetic retinopathy | 1.61 (1.34–1.94) | 5.5×10^-7^ | 1.24 (1.17–1.32) | 1.7×10^-11^ |
| Foot/toe amputation | 3.50 (2.23–5.49) | 5.1×10^-8^ | 1.41 (1.26–1.57) | 1.9×10^-9^ |

**Note.** Values are hazard ratios (HRs) with 95% confidence intervals after a 24-month landmark lag. LHS estimates are adjusted Cox-model estimates in the matched severe-deficiency (<10 ng/mL) versus sufficiency (>30 ng/mL) cohort. TriNetX estimates are from propensity-score-matched US cohorts. The TriNetX cerebrovascular estimate corresponds to ischemic cerebrovascular accident. The 12-month sensitivity analyses are shown in Supplementary Figure 2.

## Supplementary Table 6. LHS time-dependent vitamin D supplementation associations under 6-, 12-, and 24-month landmark lags

| **Outcome** | **Supplementation category (ng/mL-equivalent)** | **6-month lag HR (95% CI); P** | **12-month lag HR (95% CI); P** | **24-month lag HR (95% CI); P** |
| --- | --- | --- | --- | --- |
| All-cause mortality | 0.2-5 | 0.82 (0.79–0.84); P=2.1×10^-46 | 0.83 (0.81–0.85); P=7.6×10^-38 | 0.84 (0.81–0.86); P=2.1×10^-31 |
| All-cause mortality | 5-10 | 0.79 (0.76–0.82); P=5.5×10^-28 | 0.79 (0.76–0.83); P=1.1×10^-26 | 0.80 (0.76–0.83); P=8.8×10^-24 |
| All-cause mortality | >10 | 0.72 (0.68–0.77); P=1.0×10^-28 | 0.73 (0.69–0.77); P=1.2×10^-27 | 0.73 (0.69–0.77); P=7.2×10^-26 |
| Diabetes mellitus | 0.2-5 | 0.90 (0.87–0.92); P=2.4×10^-12 | 0.90 (0.87–0.93); P=6.8×10^-11 | 0.89 (0.86–0.92); P=8.5×10^-11 |
| Diabetes mellitus | 5-10 | 0.90 (0.85–0.96); P=0.0015 | 0.90 (0.85–0.96); P=0.002 | 0.91 (0.85–0.98); P=0.01 |
| Diabetes mellitus | >10 | 0.85 (0.77–0.93); P=3.3×10^-4 | 0.86 (0.78–0.94); P=0.0013 | 0.87 (0.79–0.96); P=0.0055 |
| Myocardial infarction | 0.2-5 | 0.87 (0.83–0.91); P=2.0×10^-8 | 0.88 (0.83–0.92); P=3.3×10^-7 | 0.88 (0.84–0.93); P=7.4×10^-6 |
| Myocardial infarction | 5-10 | 0.81 (0.73–0.89); P=3.7×10^-5 | 0.80 (0.72–0.89); P=2.2×10^-5 | 0.81 (0.73–0.90); P=1.2×10^-4 |
| Myocardial infarction | >10 | 0.85 (0.74–0.98); P=0.023 | 0.87 (0.76–1.00); P=0.044 | 0.89 (0.77–1.02); P=0.10 |
| Foot/toe amputation | 0.2-5 | 0.96 (0.82–1.13); P=0.65 | 0.98 (0.83–1.16); P=0.82 | 1.03 (0.86–1.23); P=0.76 |
| Foot/toe amputation | 5-10 | 1.04 (0.77–1.40); P=0.81 | 1.09 (0.80–1.48); P=0.58 | 1.11 (0.81–1.52); P=0.52 |
| Foot/toe amputation | >10 | 1.19 (0.79–1.78); P=0.41 | 1.22 (0.81–1.84); P=0.33 | 1.31 (0.87–1.98); P=0.20 |

**Note.** Hazard ratios compare each time-updated supplementation category with the reference category of 0–0.2 ng/mL-equivalent supplementation. Models jointly included baseline 25(OH)D category and time-dependent supplementation exposure and used the covariate adjustment described in Supplementary Methods 1. Landmark analyses retained participants alive, observable, and outcome-free at the landmark, with follow-up re-zeroed at 6, 12, or 24 months. Estimates remained materially stable for mortality, diabetes mellitus, and myocardial infarction; foot/toe amputation estimates were imprecise and showed no clear inverse supplementation association.

## Supplementary Table 7. Melanoma and non-melanoma skin-cancer subtype analyses in LHS and TriNetX

**Panel A. LHS matched severe-deficiency versus sufficiency cohort**

| **Outcome** | **6-month lag adjusted HR (95% CI); P** | **12-month lag adjusted HR (95% CI); P** | **24-month lag adjusted HR (95% CI); P** |
| --- | --- | --- | --- |
| Melanoma | 0.71 (0.46–1.11); P=0.13 | 0.69 (0.44–1.07); P=0.099 | 0.65 (0.40–1.06); P=0.084 |
| Non-melanoma skin cancer | 0.49 (0.40–0.59); P=6.4 × 10^-14 | 0.49 (0.41–0.59); P=2.6 × 10^-13 | 0.47 (0.39–0.58); P=3.0 × 10^-13 |

**Panel B. TriNetX propensity-score-matched cohort, 24-month landmark lag**

| **Outcome** | **HR (95% CI)** | **P value** |
| --- | --- | --- |
| Melanoma | 0.64 (0.56–0.73) | 1.6 × 10^-11 |
| Non-melanoma skin cancer | 0.72 (0.68–0.76) | 9.0 × 10^-34 |

**Panel C. LHS adjusted time-dependent Cox models by baseline 25(OH)D and supplementation category**

| **Outcome** | **Exposure** | **Category** | **6-month lag HR (95% CI); P** | **12-month lag HR (95% CI); P** | **24-month lag HR (95% CI); P** |
| --- | --- | --- | --- | --- | --- |
| Melanoma | Baseline 25(OH)D | 0-5 | 0.41 (0.25–0.66); P=3.1 × 10^-4 | 0.40 (0.24–0.66); P=3.3 × 10^-4 | 0.30 (0.17–0.56); P=1.1 × 10^-4 |
| Melanoma | Baseline 25(OH)D | 5-10 | 0.53 (0.44–0.65); P=9.8 × 10^-10 | 0.52 (0.43–0.64); P=8.0 × 10^-10 | 0.50 (0.40–0.63); P=1.2 × 10^-9 |
| Melanoma | Baseline 25(OH)D | 10-15 | 0.66 (0.57–0.76); P=2.2 × 10^-8 | 0.64 (0.55–0.74); P=5.1 × 10^-9 | 0.65 (0.55–0.76); P=7.5 × 10^-8 |
| Melanoma | Baseline 25(OH)D | 15-20 | 0.83 (0.73–0.95); P=0.0055 | 0.82 (0.72–0.94); P=0.0036 | 0.82 (0.71–0.94); P=0.0057 |
| Melanoma | Time-varying supplementation | 0.2-5 | 1.05 (0.92–1.19); P=0.47 | 1.06 (0.93–1.20); P=0.39 | 1.06 (0.93–1.22); P=0.39 |
| Melanoma | Time-varying supplementation | 5-10 | 1.14 (0.91–1.44); P=0.25 | 1.15 (0.91–1.45); P=0.24 | 1.15 (0.90–1.46); P=0.26 |
| Melanoma | Time-varying supplementation | >10 | 1.16 (0.85–1.58); P=0.34 | 1.20 (0.88–1.63); P=0.25 | 1.25 (0.91–1.70); P=0.17 |
| Non-melanoma skin cancer | Baseline 25(OH)D | 0-5 | 0.42 (0.33–0.52); P=2.6 × 10^-14 | 0.42 (0.34–0.53); P=1.4 × 10^-13 | 0.41 (0.33–0.53); P=5.9 × 10^-13 |
| Non-melanoma skin cancer | Baseline 25(OH)D | 5-10 | 0.58 (0.53–0.64); P=5.7 × 10^-31 | 0.58 (0.53–0.64); P=8.7 × 10^-30 | 0.58 (0.52–0.64); P=2.9 × 10^-28 |
| Non-melanoma skin cancer | Baseline 25(OH)D | 10-15 | 0.75 (0.70–0.80); P=2.5 × 10^-17 | 0.74 (0.69–0.80); P=3.7 × 10^-17 | 0.74 (0.69–0.80); P=5.6 × 10^-16 |
| Non-melanoma skin cancer | Baseline 25(OH)D | 15-20 | 0.86 (0.81–0.91); P=1.6 × 10^-6 | 0.86 (0.80–0.91); P=2.5 × 10^-6 | 0.84 (0.79–0.90); P=7.2 × 10^-7 |
| Non-melanoma skin cancer | Time-varying supplementation | 0.2-5 | 1.10 (1.04–1.17); P=0.0017 | 1.10 (1.04–1.17); P=0.0018 | 1.11 (1.04–1.18); P=0.0018 |
| Non-melanoma skin cancer | Time-varying supplementation | 5-10 | 1.17 (1.06–1.29); P=0.0023 | 1.17 (1.06–1.29); P=0.0023 | 1.18 (1.06–1.31); P=0.0018 |
| Non-melanoma skin cancer | Time-varying supplementation | >10 | 1.18 (1.03–1.35); P=0.014 | 1.19 (1.04–1.36); P=0.011 | 1.22 (1.06–1.40); P=0.0048 |

**Note.** Panel A compares severe vitamin D deficiency (<10 ng/mL) with sufficiency (>30 ng/mL) in the LHS matched cohort using adjusted Cox models. Panel B reports the corresponding TriNetX subtype estimates available for the requested 24-month landmark analysis. Panel C reports LHS time-dependent models in which baseline 25(OH)D categories (reference >20 ng/mL) and supplementation categories (reference 0–0.2 ng/mL-equivalent) were modeled jointly. Melanoma comprised CA SKIN records with ICD-9-CM codes beginning 172; non-melanoma skin cancer comprised CA SKIN records with codes beginning 173.

## Supplementary Figure 1. Flowchart of Matched Vitamin D Cohort Construction and Matching

**Legend:**
Flowchart illustrating the construction of a matched cohort comparing individuals with severe vitamin D deficiency (<10 ng/mL) to those with sufficient levels (≥30 ng/mL) in the Leumit Health Services (LHS) database. Adults aged ≥30 years with at least one vitamin D test 2009-2020 were eligible. Individuals were categorized based on their calendar-adjusted serum 25(OH)D levels.
Only those over 30 with available BMI data were included for matching. A 1:1 nearest-neighbor matching was performed based on age, sex, smoking status, BMI, and BP categories, socioeconomic status (SES), ethnicity, and year of LHS enrollment yielding 12,352 matched pairs.

## Supplementary Figure 2. Sensitivity analyses performed with a 12-month lag
